# Risk of sudden cardiac arrest and ventricular arrhythmia with sulfonylureas: An experience with conceptual replication in two independent populations

**DOI:** 10.1038/s41598-020-66668-5

**Published:** 2020-06-22

**Authors:** Neil Dhopeshwarkar, Colleen M. Brensinger, Warren B. Bilker, Samantha E. Soprano, James H. Flory, Ghadeer K. Dawwas, Joshua J. Gagne, Sean Hennessy, Charles E. Leonard

**Affiliations:** 1 Center for Pharmacoepidemiology Research and Training, Department of Biostatistics, Epidemiology, and Informatics, Perelman School of Medicine, University of Pennsylvania, Philadelphia, Pennsylvania USA; 2 Endocrinology Service, Department of Subspecialty Medicine, Memorial Sloan Kettering Cancer Center, New York, New York USA; 3 Division of Pharmacoepidemiology and Pharmacoeconomics, Department of Medicine, Brigham and Women’s Hospital and Harvard Medical School, Boston, Massachusetts USA; 4 Department of Systems Pharmacology and Translational Therapeutics, Perelman School of Medicine, University of Pennsylvania, Philadelphia, Pennsylvania USA

**Keywords:** Cardiology, Endocrinology, Health care, Medical research, Risk factors

## Abstract

Sulfonylureas are commonly used to treat type 2 diabetes mellitus. Despite awareness of their effects on cardiac physiology, a knowledge gap exists regarding their effects on cardiovascular events in real-world populations. Prior studies reported sulfonylurea-associated cardiovascular death but not serious arrhythmogenic endpoints like sudden cardiac arrest (SCA) or ventricular arrhythmia (VA). We assessed the comparative real-world risk of SCA/VA among users of second-generation sulfonylureas: glimepiride, glyburide, and glipizide. We conducted two incident user cohort studies using five-state Medicaid claims (1999–2012) and Optum Clinformatics commercial claims (2000–2016). Outcomes were SCA/VA events precipitating hospital presentation. We used Cox proportional hazards models, adjusted for high-dimensional propensity scores, to generate adjusted hazard ratios (aHR). We identified 624,406 and 491,940 sulfonylurea users, and 714 and 385 SCA/VA events, in Medicaid and Optum, respectively. Dataset-specific associations with SCA/VA for both glimepiride and glyburide (vs. glipizide) were on opposite sides of and could not exclude the null (glimepiride: aHR_Medicaid_ 1.17, 95% CI 0.96–1.42; aHR_Optum_ 0.84, 0.65–1.08; glyburide: aHR_Medicaid_ 0.87, 0.74–1.03; aHR_Optum_ 1.11, 0.86–1.42). Database differences in data availability, populations, and documentation completeness may have contributed to the incongruous results. Emphasis should be placed on assessing potential causes of discrepancies between conflicting studies evaluating the same research question.

## Introduction

Second-generation sulfonylureas are a mainstay of type 2 diabetes mellitus (T2DM) management. Although their use is slowly declining^1^, this pharmacologic class remains the most common add-on to metformin^2,3^. Cardiovascular effects of sulfonylureas continue to be debated. Findings from the recently completed *Cardiovascular Outcome Trial of Linagliptin Versus Glimepiride in Type 2 Diabetes* (CAROLINA) trial^4^ may eventually assuage concerns of glimepiride-associated all-cause and cardiovascular death^5^ reported in prior clinical trials and meta-analyses^6–10^, but little is known of cardiovascular safety among users of other sulfonylureas. Further, despite pre-existing data on sulfonylureas’ effects on cardiac physiology^11–14^, neither CAROLINA nor *Thiazolidinediones or Sulfonylureas Cardiovascular Accidents Intervention Trial* (TOSCA.IT) provided, and *Glycemia Reduction Approaches in Diabetes* (GRADE) will not provide data on serious arrhythmogenic endpoints like sudden cardiac arrest (SCA) and ventricular arrhythmia (VA). Thus, there exists a major knowledge gap in the arrhythmogenic effects of individual second-generation sulfonylureas. We therefore set forth to assess the real-world comparative safety of glyburide, glimepiride, and glipizide by conducting two incident user cohort studies—in independent United States (US) populations of Medicaid and commercial health insurance beneficiaries—to elucidate associations with SCA and VA.

Our decision to ask this clinical question in independent healthcare claims datasets acknowledges the importance of replicability in pharmacoepidemiology^15–18^. A historic lack of transparency in reporting detailed study methods and key operational decisions has hindered direct (i.e., reproducing a study result using the same database) and conceptual (i.e., reproducing a study finding using a different database and subsequently a different population) replication^16^ and confused decision makers. We focused on conceptual replication because of its broader impact and potential to advance the T2DM evidence base^16^. Yet, conceptual replication can be challenging because of inherent differences in populations under study and/or databases used; data source choice can substantively affect results of nonexperimental population-based studies^19^. While examples of both randomized and nonrandomized studies with the same research question but conflicting results abound^20–24^, few have assessed probable causes of their inconsistencies. We therefore evaluated the same clinical question in independent populations of second-generation sulfonylurea users, compared this to our prior results on the topic^25^, and explored potential reasons for similarities and differences among findings.

## Results

### Baseline characteristics

We identified 268,094 glipizide users, 124,354 glimepiride users, and 231,958 glyburide users in Medicaid and 206,034, 151,229, and 134,677 users in Optum (Supplementary Tables 1 and 2). Users were similar in age across datasets (median = 57.8 years in Medicaid, 58.2 years in Optum) (Table 1). The majority of users in Medicaid (59.8%), but not Optum (43.9%), were female. Users were largely of white race in both Optum (56.9%) and Medicaid (34.6%). Substantial proportions of users had diagnoses of hypertension (57.9% in Medicaid, 60.5% in Optum), dyslipidemia (42.5% in Medicaid, 56.7% in Optum), ischemic heart disease (20.9% in Medicaid, 14.3% in Optum), and depression (24.6% in Medicaid, 13.9% in Optum). Small proportions had pre-existing cardiomegaly (5.8% in Medicaid, 3.0% in Optum), cardiac conduction disorders (2.0% in Medicaid, 1.6% in Optum), and congenital heart anomalies (1.3% in Medicaid, 0.4% in Optum). Episodes of serious hypoglycemia during baseline were uncommon (2.2% in Medicaid, 0.6% in Optum).

**Table 1:** Characteristics of second-generation sulfonylurea users in Medicaid and Optum.

		Medicaid	Optum
Users, N		624,406	491,940
Person-years of follow-up, sum, among all users		201,183	197,848
Days of follow-up, median (5^th^, 95^th^ percentile), per user		46 (1, 428)	76 (3, 532)
Proportion of follow-up time covered by days’ supply of sulfonylurea dispensings, median (5^th^, 95^th^ percentile)		87.1 (67.4, 100.0)	88.3 (67.4, 100.0)
**Demographics**	**Group**	**% (unless otherwise noted)**	
Age, in years, at cohort entry	Median (Q1-Q3)	57.8 (48.1–67.1)	58.2 (49.8–66.8)
Sex	Female	59.8	43.9
Race/Ethnicity	White	34.6	56.9
	Black	18.7	13.3
	Hispanic/Latino	23.7	15.4
	Asian	6.4	3.7
	Unknown/Missing/Other	16.6	10.7
State of residence	CA	45.0	unavailable
	FL	11.9	
	NY	27.1	
	OH	8.8	
	PA	7.2	
Census level	New England	0.0	2.6
	Middle Atlantic	34.3	4.8
	East North Central	8.8	13.1
	West North Central	0.0	8.1
	South Atlantic	11.9	27.6
	East South Central	0.0	4.7
	West South Central	0.0	16.5
	Mountain	0.0	6.8
	Pacific	45.0	15.2
	Unknown	0.0	0.6
Education level	Less than 12th Grade	unavailable	1.9
	High School Diploma		37.4
	Less than Bachelor Degree		44.9
	Bachelor Degree Plus		7.7
	Unknown		8.1
Housing	Probable Homeowner	unavailable	69.1
	Unknown		30.9
Household income	<$40K	unavailable	18.1
	$40K-$49K		6.9
	$50K-$59K		6.6
	$60K-$74K		8.5
	$75K-$99K		10.8
	$100K +		16.9
	Unknown		32.1
Total net worth of the primary customer	<$25K	unavailable	11.3
	$25K-$149K		21.3
	$150K-$249K		14.5
	$250K-$499K		21.6
	$500K +		14.5
	Unknown		16.8
Calendar year of cohort entry^‡^	2000	6.3	-
	2001	7.6	3.3
	2002	7.8	4.1
	2003	7.6	3.9
	2004	6.3	4.1
	2005	8.1	12.6
	2006	11.2	6.9
	2007	7.6	6.2
	2008	6.3	7.4
	2009	7.1	6.4
	2010	8.4	6.6
	2011	7.4	6.9
	2012	8.3	6.8
	2013	unavailable	6.5
	2014		5.6
	2015		6.0
	2016		6.5
Medicare enrolled	Yes	48.7	30.5
Nursing home residence ever during baseline	Yes	6.0	1.3
Healthcare use intensity measures, in baseline period*	**Group**	**Measure of central tendency**	
No. prescriptions dispensed, total	Median (Q1-Q3)	37.0 (9.0–78.0)	19.0 (4.0–41.0)
No. prescriptions dispensed, by unique drug	Median (Q1-Q3)	11.0 (5.0–19.0)	6.0 (3.0–11.0)
No. outpatient diagnosis codes, total	Median (Q1-Q3)	29.0 (10.0–69.0)	19.0 (6.0–40.0)
No. outpatient diagnosis codes, by unique code	Median (Q1-Q3)	11.0 (5.0–21.0)	10.0 (4.0–16.0)
No. outpatient CPT-4/HCPCS codes, total	Median (Q1-Q3)	34.0 (12.0–78.0)	21.0 (7.0–42.0)
No. outpatient CPT-4/HCPCS codes, by unique code	Median (Q1-Q3)	21.0 (9.0–40.0)	14.0 (6.0–26.0)
**Other investigator pre-defined covariates, in baseline period**	**Group**	**%**	
Disorders of lipid metabolism	Yes	42.5	56.7
Rheumatic heart disease, chronic	Yes	2.3	1.2
Hypertensive disease	Yes	57.9	60.5
Ischemic heart disease	Yes	20.9	14.3
Conduction disorders	Yes	2.0	1.6
Heart failure/cardiomyopathy	Yes	12.8	5.9
Cardiomegaly	Yes	5.8	3.0
Congenital anomalies of the heart, other	Yes	1.3	0.4
Implantable cardioverter defibrillator/pacemaker use	Yes	0.9	0.8
Kidney disease	Yes	16.2	13.6
Depression	Yes	24.6	13.9
Obesity	Yes	11.7	14.1
Tobacco use	Yes	8.6	8.9
Alcohol abuse	Yes	3.3	1.3
Hypoglycemia, serious	Yes	2.2	0.6
Diabetes mellitus, type 2^†^	Yes	95.1	97.9
Adapted Diabetes Complications Severity Index	0	50.7	61.1
	1	12.5	14.1
	2	14.6	12.0
	3	6.8	5.1
	4	5.8	3.5
	5+	9.5	4.1
Drugs in the 30 days prior to cohort entry**	**Group**	**%**	
alpha-glucosidase inhibitor	Yes	0.3	0.1
amylin analog	Yes	0.0	0.0
dipeptidyl peptidase-4 inhibitor	Yes	1.9	3.6
glucagon-like peptide-1 receptor agonist	Yes	0.2	1.0
insulin	Yes	7.8	3.8
metformin	Yes	22.3	24.4
meglitinide	Yes	1.0	0.4
sodium-glucose co-transporter 2 inhibitor^§^	Yes	0.0	0.3
thiazolidinedione	Yes	7.8	5.5
CYP2C9 inhibitor	Yes	4.8	2.7
CYP3A4 inhibitor	Yes	3.7	2.1
CYP2C9 inducer	Yes	0.9	0.2
CYP3A4 inducer	Yes	6.2	3.7
drug with known risk of TdP^§§^	Yes	9.0	6.3
drug with known, possible, or conditional risk of TdP^§§^	Yes	44.9	32.1
≥ 5 prescription dispensings for unique drugs	Yes	39.9	21.7
**Laboratory covariates, in baseline period**	**Group**	**%**	
Blood glucose	No lab ever in past	unavailable	51.2
	No lab in 1-year baseline		23.5
	Normal		8.2
	Low abnormal		0.2
	High abnormal		16.9
Hemoglobin A1C	No lab ever in past		51.2
	No lab in 1-year baseline		26.0
	Normal		6.0
	Low abnormal		0.0
	High abnormal		16.8
Serum creatinine	No lab ever in past		51.2
	No lab in 1-year baseline		23.3
	Normal		19.5
	Low abnormal		3.4
	High abnormal		2.5
Hematocrit	No lab ever in past		51.2
	No lab in 1-year baseline		30.4
	Normal		16.2
	Low abnormal		1.9
	High abnormal		0.2
Hemoglobin	No lab ever in past		51.2
	No lab in 1-year baseline		30.5
	Normal		15.7
	Low abnormal		2.2
	High abnormal		0.4
Hypoglycemia, laboratory measured, alert value	No lab ever in past		51.2
	No lab in 1-year baseline		23.5
	No alert value		24.8
	Alert value		0.5
Hypoglycemia, laboratory measured, clinically significant value	No lab ever in past		51.2
	No glucose in 1-year baseline		23.5
	No clinically significant value		25.2
	Clinically significant value		0.2

CA = California; CPT = Current Procedural Terminology; CYP = cytochrome P450; FL = Florida; HCPCS = Healthcare Common Procedure Coding System; ICD = International Classification of Diseases; NY = New York; OH = Ohio; PA = Pennsylvania; Q = quartile; TdP = torsade de pointes

See Supplementary Table 1 and Supplementary Table 2 for intra-database comparisons of characteristics by individual sulfonylurea.

*The following healthcare utilization covariates were excluded from presentation in the table, as their median values were zero for each sulfonylurea: ^#^inpatient ICD-9 diagnosis codes; ^#^unique inpatient ICD-9 diagnosis codes; ^#^inpatient ICD-9 procedure codes; ^#^unique inpatient ICD-9 procedure codes; ^#^inpatient CPT/HCPCS procedure codes; ^#^unique inpatient CPT/HCPCS procedure codes; ^#^outpatient ICD-9 procedure codes; ^#^unique outpatient ICD-9 procedure codes; ^#^other setting ICD-9 diagnosis codes; ^#^unique other setting ICD-9 diagnosis codes; ^#^other setting ICD-9 procedure codes; ^#^unique other setting ICD-9 procedure codes; ^#^laboratory LOINC codes (Optum only); ^#^unique laboratory LOINC codes (Optum only).

**Antimicrobial drugs in each category were examined within 14 (rather than 30) days prior to cohort entry; these agents are typically prescribed for acute conditions.

^†^Defined by ratio of type 1 (e.g., ICD-9 250.X1 or 250.X3) to type 2 (e.g., ICD-9 250.X0 or 250.X2) codes ≤0.5, ascertained during baseline and on cohort entry date.

^‡^Prespecified covariate not forced into propensity score, but included as a categorical variable in outcome model.

^§^Not marketed during years of study in Medicaid analysis.

^§§^Per CredibleMeds (AZCERT Inc.: Oro Valley, AZ).

### Follow-up time and crude incidence rates

Among 201,183 person-years (p-y) of follow-up in Medicaid (median follow-up of 46 days per user), we identified 714 SCA/VAs (crude incidence = 3.55 per 1,000 p-y), 375 (52.5%) of which were fatal. Among 197,848 p-y of follow-up in Optum (median follow-up of 76 days per user), we identified 385 SCA/VAs (1.95 per 1,000 p-y). In secondary analyses restricted to the first 30 days of follow-up, we identified 325 (7.52 per 1,000 p-y) and 110 SCA/VAs (3.00 per 1,000 p-y) in Medicaid and Optum respectively.

### Modeled findings

The propensity score included 482 covariates in Medicaid (55 predefined and 427 empirically identified by the high-dimensional propensity score [hdPS] algorithm) and 529 covariates in Optum (70 predefined and 459 empirically identified by the hdPS algorithm) (Supplementary Tables 3–5). Crude and adjusted hazard ratios (aHRs) are presented in Table 2. Notably, dataset-specific associations between glimepiride (vs. glipizide) and SCA/VA were on opposite sides of and could not exclude the null (aHR_Medicaid_ 1.17, 95% confidence interval [CI] 0.96–1.42; aHR_Optum_ 0.84, 0.65–1.08). Similarly, dataset-specific associations between glyburide (vs. glipizide) and SCA/VA were on opposite sides of and could not exclude the null (aHR_Medicaid_ 0.87, 0.74–1.03; aHR_Optum_ 1.11, 0.86–1.42). In the Medicaid-only analysis of the secondary outcome, glimepiride (vs. glipizide) was associated with an elevated risk of sudden cardiac death (SCD)/fatal VA (aHR 1.33, 1.02–1.75). Results from secondary analyses were generally consistent with the primary analysis (Supplementary Table 6) and we identified no dose-response relationships (Supplementary Table 7).

**Table 2:** Outcomes, incidence rates, and effect estimates for primary analysis.

	Medicaid			Optum		
**Outcomes during follow-up**	**N**					
SCA/VA	714			385		
SCD/fatal VA	375			—		
**Measure of outcome occurrence**	**Crude incidence rate, per 1,000 person-years (95% confidence interval)**					
SCA/VA	3.55 (3.29–3.82)			1.95 (1.76–2.15)		
SCD/fatal VA	1.86 (1.68–2.06)			—		
**Relative effect estimates for outcome**	**Hazard Ratio (95% confidence interval)**					
	**Glipizide**	**Glimepiride**	**Glyburide**	**Glipizide**	**Glimepiride**	**Glyburide**
**SCA/VA**						
Unadjusted^*^	1.00 (reference)	0.96 (0.79–1.16)	0.78 (0.66–0.93)	1.00 (reference)	0.75 (0.59–0.96)	0.94 (0.74–1.20)
hdPS-adjusted^**^	1.00 (reference)	1.17 (0.96–1.42)	0.87 (0.74–1.03)	1.00 (reference)	0.84 (0.65–1.08)	1.11 (0.86–1.42)
**SCD/fatal VA**						
Unadjusted^†^	1.00 (reference)	1.03 (0.79–1.34)	0.83 (0.66–1.05)	—	—	—
hdPS-adjusted^‡^	1.00 (reference)	1.33 (1.02–1.75)	0.91 (0.72–1.20)	—	—	—

hdPS = high-dimensional propensity score; SCA = sudden cardiac arrest; SCD = sudden cardiac death; VA = ventricular arrhythmia.

*Failed a test for non-proportional hazards in Medicaid (p = 0.034), but not in Optum (p = 0.566).

**Did not fail a test for non-proportional hazards in Medicaid (p = 0.052) or Optum (p = 0.658).

^†^Did not fail a test for non-proportional hazards (p = 0.213).

^‡^Did not fail a test for non-proportional hazards (p = 0.265).

## Discussion

This study examined the comparative risk of SCA/VA among users of individual second-generation sulfonylureas in two independent US populations. The crude incidence rates of SCA/VA in the Medicaid (3.55 per 1,000 p-y) and Optum (1.95 per 1,000 p-y) populations are similar to those reported in other diabetic populations^26,27^ and higher than those reported in general populations^27,28^, potentially explained by the two to fourfold increase in risk of SCA with diabetes^26,29^. Although analyses of both populations found non-statistically significant differences in the risk of SCA/VA among users of individual second-generation sulfonylureas, the effect estimates were on opposite sides of the null. These results demonstrate the sensitivity of study findings to the specific data source used despite using similar study methods, and the importance of the conceptual replication of findings in multiple populations.

Differences in data availability between databases are an obvious potential source of discordant results. There were two notable data dimensions available in Optum but not in Medicaid. First, Optum provides laboratory results for a subset of beneficiaries. In order to more robustly characterize baseline T2DM severity and SCA/VA risk for Optum beneficiaries, we pre-specified relevant laboratory values to be included in the propensity scores (e.g., blood glucose, HbA1c, serum creatinine, hematocrit, and hemoglobin). Furthermore, we incorporated laboratory values as a data dimension in the hdPS algorithm, allowing for the empirical selection of laboratory values as potential proxies for unmeasured confounders. Laboratory findings were unavailable in Medicaid, and thus were not included as pre-specified or empirically identified covariates in the propensity score estimation. To understand the effect of incorporating laboratory results in the propensity score, we performed a post hoc sensitivity analysis in Optum in which we estimated the propensity score without pre-specified and empirically identified laboratory covariates. The study results were consistent with the primary analysis, suggesting that discrepancies between the Medicaid and Optum study findings were due to other causes. Second, socioeconomic variables (education level, housing, household income, and net worth of primary customer) were available for Optum but not Medicaid beneficiaries. We performed a post hoc sensitivity analysis removing these covariates (in addition to laboratory covariates) from the propensity score estimation; the results were unchanged.

Death dates were available for Medicaid but not Optum beneficiaries. This limited censoring on death to Medicaid analyses. Optum beneficiaries who died (for reasons other than the outcome of interest) would likely have been censored on their disenrollment date. This may be evident in differences in censoring reasons by dataset; censoring due to disenrollment occurred in 12.7% of Optum vs. 6.6% of Medicaid beneficiaries, though disenrollment for some beneficiaries may have been caused by reasons other than death. Optum beneficiaries under study may have had a lag between dates of death and disenrollment (e.g., if benefits terminate at the end of the calendar month in which a beneficiary dies). This may be reflected in longer observed median follow-up times among Optum vs. Medicaid beneficiaries (76 vs. 46 days). Exposure misclassification would be present during this lag period, since expired individuals cannot be sulfonylurea-exposed. This misspecification could have resulted in longer follow-up times and subsequently underestimated incidence rates of the primary outcome among Optum beneficiaries.

Each database represents a distinct underlying population with different characteristics that may contribute to disparate findings. Optum comprises privately-insured, employed individuals across the US, while Medicaid comprises publicly-insured low-income adults, elders, children, pregnant women, and persons with disabilities^30^. Thus, Medicaid beneficiaries tend to have more comorbidities and be more socioeconomically disadvantaged and overall vulnerable compared to commercially insured beneficiaries. Clinical characteristics of the two study populations indicate this; sulfonylurea users in Medicaid (vs. Optum) were more likely to have ischemic heart disease (20.9% vs. 14.3%), heart failure/cardiomyopathy (12.8% vs. 5.9%), cardiomegaly (5.8% vs. 3.0%), kidney disease (16.2% vs. 13.6%), and prior episodes of serious hypoglycemia (2.2% vs. 0.6%). These conditions are recognized as determinants of SCA risk in persons with DM^11^, therefore the dissimilar profiles of users may portend differential imbalance in unmeasured confounders and subsequently residual confounding. However, this should have been forestalled by use of data-adaptive hdPS methods.

International Classification of Diseases, 9^th^ Revision, Clinical Modification (ICD-9-CM) codes are commonly used to measure outcomes in healthcare database studies but have varying ability to validly capture the diagnoses they represent^31^. In order to most accurately capture our primary outcome, we used a validated algorithm for identifying outpatient-originating SCA/VA events. This algorithm was validated against medical records in a Medicaid population resulting in an overall positive predictive value (PPV) of 85.3%, and event-specific PPVs of 92.3% and 74.4% for SCA and VA respectively^32^. We can expect this algorithm to perform similarly in our Medicaid study but its transportability to a commercial claims dataset like Optum is undetermined, as the algorithm’s specificity and sensitivity is unknown. Since the algorithm’s overall PPV can change with a varied outcome prevalence or a varied distribution of outcome-defining events (i.e., proportion of SCA events vs. VA events), the algorithm may perform differently in Optum. In fact, both overall outcome prevalence (0.08% vs. 0.11%) and proportion of outcome-defining SCA events (59.7% vs. 79.0%) differed in Optum compared to Medicaid, suggesting an altered PPV for identifying SCA/VA in Optum. Varied PPVs between the two databases could have led to differing rates of outcome misclassification, a potential contributor to discrepant results. Additionally, the International Classification of Diseases, 10^th^ Revision, Clinical Modification (ICD-10-CM) component of the algorithm, used in the Optum but not Medicaid analysis, has not been validated and may have contributed to a varied PPV. However, only 9% of total events in Optum were captured using ICD-10-CM codes, suggesting that this may not have been a principal driver of the discrepancy.

Other potential reasons for incongruous findings may be subtle; thus, assessing their impact is difficult. Formulary considerations, for example, could affect results if medication coverage restrictions were made for health-related reasons. Among second-generation sulfonylurea users under study, there were notably fewer glyburide users in Optum (27.4%) vs. Medicaid (37.1%). This imbalance may have been driven by differing coverage restrictions on glyburide between insurance plans, possibly due to glyburide’s increased comparative risk of serious hypoglycemia among second-generation sulfonylurea agents in elderly patients^33^. Such a restriction could have resulted in a channeling effect in which a specific subset of beneficiaries was less likely to be exposed to glyburide. Evidence of such formulary considerations, however, is not directly attainable from the data.

Notwithstanding differences in availability of laboratory, socioeconomic, and death data, Medicaid and Optum both provided data on medical diagnoses, procedures, and medication dispensings, allowing for similar methodologic approaches to be used in the two studies. Nonetheless, there may be differences in documentation completeness within each of these data dimensions, as only health services billed to the particular insurance plan were recorded. If beneficiaries of one insurance plan had fewer health services billed to that insurance (e.g., beneficiaries using secondary insurance coverage or paying out-of-pocket) than beneficiaries of the other plan, there would be differential capture of healthcare information between the two databases.

We previously examined the comparative risk of SCA/VA with individual second-generation sulfonylureas in 1999–2010 Medicaid data using similar methods^25^. Results were generally consistent with the current Medicaid study containing two more years of data, with only two minor differences. First, the primary outcome effect estimate for glyburide shifted slightly towards the null (aHR 0.82, 0.69–0.98 in prior study vs. 0.87, 0.74–1.03 in current study), no longer reaching statistical significance in the current study. This may have been due to differences in variables included in the propensity score model (e.g., the inclusion of the adapted Diabetes Complications Severity Index in the current but not prior study) or changes in prescribing patterns and confounding by indication introduced by the additional two years of data. Second, the secondary outcome effect estimate for glimepiride reached statistical significance in the current study but not in the prior study—potentially due to the increase in sample size. The general agreement between the two Medicaid studies further supports that discrepancies in the current findings may be attributed to differences between databases rather than issues with methodologic reproducibility.

Our studies have limitations. First, a lack of access to biosamples prevented examination of genetic determinants of SCA/VA risk. Second, adjustment for family history of diseases was under-ascertained due to reliance on diagnostic coding. Third, sulfonylurea exposure was defined using prescription dispensings and may not reflect ingestion. To partly address this, we conducted sensitivity analyses in which we modified grace periods between prescriptions. Fourth, outcomes may have been under-ascertained due to the inability to capture fatal events that did not result in hospital presentation, potentially biasing effect estimates towards the null. However, prior work suggests that 69–80% of persons experiencing an out-of-hospital cardiac arrest^34,35^ and up to 88% of persons experiencing a witnessed ventricular tachycardia survive to hospital admission^36^, although recent registry data from Cardiac Arrest Registry to Enhance Survival (CARES) suggests poorer survival-to-admission rates (18–49%, depending on presenting characteristics)^37^. Lastly, although we explored potential reasons for discrepancies in findings between databases, we were unable to directly confirm these effects on study results.

Healthcare claims databases contain both obvious and subtle differences in data availability, population characteristics, and documentation completeness. Our results demonstrate the potential impact of these differences on study findings, despite similarity in study design and analytic methodology. With a growing emphasis on the replication of pharmacoepidemiologic study findings in multiple datasets to inform regulatory decision making and clinical practice, future studies investigating the same clinical question may arrive at differing conclusions. Rather than discount these results, investigators should focus more effort on assessing the specific causes of discrepancies to better elucidate study findings.

## Methods

### Overview and study populations

We conducted two hdPS-adjusted cohort studies to determine rates of SCA/VA among second-generation sulfonylurea users aged 30–75 years. We excluded younger users because SCA/VA is rare and unlikely due to prescription drugs in this population^38^, and older users because competing comorbidities may mimic SCA/VA. The cohorts consisted exclusively of person-time exposed to glimepiride, glipizide, or glyburide. Data sources included demographic, enrollment, and healthcare claims from: 1) 1999–2012 Medicaid programs of California, Florida, New York, Ohio, and Pennsylvania (~40% of the national Medicaid population), supplemented with Medicare claims for dual-enrollees, and linked to the Social Security Administration Death Master File; and 2) 2000–2016 Optum Clinformatics commercial health insurance data, which includes >71 million commercially insured and Medicare Advantage beneficiaries of the largest US-based private health insurer by market share^39^. Optum, but not Medicaid, included laboratory results for a subset of individuals. Medicaid, but not Optum, included death dates.

### Defining the cohort

We defined cohort entry upon incident use of a second-generation sulfonylurea, i.e., we required a preceding 12-month baseline period devoid of any sulfonylurea dispensing. We excluded observations with the following baseline events: (1) interruption in insurance plan enrollment; (2) any-setting SCA or VA diagnosis (broader than the outcome definition below, to minimize the inclusion of recurrent events); and (3) pregnancy, to avoid channeling bias (pregnant sulfonylurea-treated patients almost exclusively receive glyburide)^40^. We included only the first observation meeting these criteria, per user.

Follow-up began at cohort entry and continued until the first occurrence of a/an: (1) outcome (defined below); (2) diagnosis suggestive of the outcome, but otherwise failing to meet the outcome definition; (3) death (Medicaid only); (4) >15-day therapy gap for the cohort-defining sulfonylurea; (5) dispensing of a sulfonylurea different than that defining cohort entry (i.e., switching); 6) dispensing of a drug with a known risk of torsade de pointes^41^; (7) insurance plan disenrollment; (8) pregnancy; or (9) the end of the dataset. We did not censor upon a non-outcome hospitalization, but excluded hospitalized time from follow-up to minimize immeasurable time bias^42^.

### Exposure and covariate ascertainments

We defined exposure by the second-generation sulfonylurea (glimepiride, glipizide, or glyburide) dispensed on the cohort entry date. We excluded sulfonylurea-unexposed individuals from the study. This allowed for direct comparisons between the three exposures of interest, and minimized the potential for selection bias and confounding by indication and other unmeasured patient characteristics, further improving the performance of hdPS^43,44^. We pre-specified glipizide as an active comparator referent, as animal models showed that it lacks a direct effect on myocardial contractility^45–49^.

Potential confounders included pre-specified variables and those identified via empiric methods, both of which informed the propensity score. Pre-specified variables included demographics, putative risk factors for SCA/VA, and measures of intensity of health care utilization (e.g., numbers of prescription drugs dispensed)^50^. Empiric variables included those identified during baseline via a high-dimensional data-adaptive approach^51,52^ which ranks and selects potential confounders based on their empiric associations with the exposure and outcome as described below.

### Outcome ascertainment

The primary outcome was an incident outpatient-originating SCA/VA event precipitating hospital presentation. Outcomes were identified in emergency department or inpatient claims having at least one discharge diagnosis code of interest in the principal or first-listed position (indicative of the reason for presentation/admission) (Supplementary Table 8). The ICD-9-CM component of this algorithm was validated against primary medical records in a Medicaid population and had a PPV of ~85% for identifying outpatient-originating SCA/VA not due to extrinsic causes^32^. We did not study inpatient-originating SCA/VA because: (1) sulfonylureas are rarely used in the hospital setting; (2) in-hospital arrhythmogenic events are often attributable to causes other than ambulatory drug exposures; and (3) neither Medicaid nor Optum record inpatient drug exposures. The secondary outcome was the subset of primary outcomes that were fatal, i.e., SCA/VA events in which the person died on the day of or the day after the event occurrence (Medicaid only).

### Statistical analysis

We calculated descriptive statistics for baseline variables, crude incidence rates, and unadjusted association measures, the latter via Cox proportional hazards models. We utilized a semi-automated, data-adaptive hdPS approach—an algorithm identifying proxies for important confounders^53^—to reduce the impact of measured and unmeasured potential confounders. We used pairwise hdPS to identify potential confounders for each sulfonylurea of interest vs. glipizide (the referent) and included all such empirically identified variables (and pre-specified variables) in a multinomial propensity score model. We first identified the 200 most prevalent diagnosis, procedure, and drug codes in each identified dimension to assess their associations with the sulfonylurea of interest (vs. glipizide) and with the outcome. We then used these associations to select the top 500 codes with the largest potential for confounding. Then, the union of all confounders arising from the two sets of 500 hdPS-identified variables (one for each sulfonylurea of interest vs. glipizide) was included in the multinomial PS, modeled using multinomial logistic regression^53^. Differences in selected covariates by exposure group were assessed using weighted conditional standardized differences^54^. We included propensity score and calendar year of cohort entry in each Cox proportional hazards regression outcome model, then calculated PS-adjusted conditional HRs and 95% CIs. Analyses were conducted using SAS version 9.4 (SAS Institute, Cary, NC). The institutional review board of the University of Pennsylvania approved this research.

## Supplementary information

Supplementary information.

## Data Availability

The data that support the findings of these studies are available from the US Centers for Medicare and Medicaid Services (CMS) and Optum Inc., but restrictions apply to the availability of these data, which were used under license for the current studies and so are not publicly available. Data may be available from the authors upon reasonable request and with explicit permission from CMS and Optum Inc.

## References

[1] American Diabetes A (2019). 9. Pharmacologic Approaches to Glycemic Treatment: Standards of Medical Care in Diabetes-2019. Diabetes Care.

[2] Kantor ED, Rehm CD, Haas JS, Chan AT, Giovannucci EL (2015). Trends in Prescription Drug Use Among Adults in the United States From 1999-2012. JAMA.

[3] Lipska KJ (2017). Trends in Drug Utilization, Glycemic Control, and Rates of Severe Hypoglycemia, 2006-2013. Diabetes Care.

[4] Rosenstock, J. *et al*. Effect of Linagliptin vs Glimepiride on Major Adverse Cardiovascular Outcomes in Patients With Type 2 Diabetes: The CAROLINA Randomized Clinical Trial. *JAMA*, 10.1001/jama.2019.13772 (2019). 10.1001/jama.2019.13772 PMC6763993 31536101

[5] Wexler, D. J. Sulfonylureas and Cardiovascular Safety: The Final Verdict? *JAMA*, 10.1001/jama.2019.14533 (2019). 10.1001/jama.2019.14533 31536110

[6] Varvaki Rados D, Catani Pinto L, Reck Remonti L, Bauermann Leitao C, Gross JL (2016). The Association between Sulfonylurea Use and All-Cause and Cardiovascular Mortality: A Meta-Analysis with Trial Sequential Analysis of Randomized Clinical Trials. PLoS Med.

[7] Pladevall M (2016). Cardiovascular risk associated with the use of glitazones, metformin and sufonylureas: meta-analysis of published observational studies. BMC Cardiovasc Disord.

[8] Monami M, Genovese S, Mannucci E (2013). Cardiovascular safety of sulfonylureas: a meta-analysis of randomized clinical trials. Diabetes Obes Metab.

[9] Ahren B (2014). HARMONY 3: 104-week randomized, double-blind, placebo- and active-controlled trial assessing the efficacy and safety of albiglutide compared with placebo, sitagliptin, and glimepiride in patients with type 2 diabetes taking metformin. Diabetes Care.

[10] Del Prato S, Camisasca R, Wilson C, Fleck P (2014). Durability of the efficacy and safety of alogliptin compared with glipizide in type 2 diabetes mellitus: a 2-year study. Diabetes Obes Metab.

[11] Leonard CE (2017). Pro- and Antiarrhythmic Actions of Sulfonylureas: Mechanistic and Clinical Evidence. Trends Endocrinol Metab.

[12] Schotborgh CE, Wilde AA (1997). Sulfonylurea derivatives in cardiovascular research and in cardiovascular patients. Cardiovasc Res.

[13] Brady PA, Terzic A (1998). The sulfonylurea controversy: more questions from the heart. J Am Coll Cardiol.

[14] Stahn A (2014). Relationship between hypoglycemic episodes and ventricular arrhythmias in patients with type 2 diabetes and cardiovascular diseases: silent hypoglycemias and silent arrhythmias. Diabetes Care.

[15] Peng RD, Dominici F, Zeger SL (2006). Reproducible epidemiologic research. Am J Epidemiol.

[16] Wang SV (2017). Reporting to Improve Reproducibility and Facilitate Validity Assessment for Healthcare Database Studies V1.0. Pharmacoepidemiol Drug Saf.

[17] Wang SV (2016). Transparency and Reproducibility of Observational Cohort Studies Using Large Healthcare Databases. Clin Pharmacol Ther.

[18] Hernan MA, Wilcox AJ (2009). Epidemiology, data sharing, and the challenge of scientific replication. Epidemiology.

[19] Madigan D (2013). Evaluating the impact of database heterogeneity on observational study results. Am J Epidemiol.

[20] Lockhart PB (2012). Periodontal disease and atherosclerotic vascular disease: does the evidence support an independent association?: a scientific statement from the American Heart Association. Circulation.

[21] Dodd S (2011). Debating the evidence: oral contraceptives containing drospirenone and risk of blood clots. Curr Drug Saf.

[22] Juni P (2004). Risk of cardiovascular events and rofecoxib: cumulative meta-analysis. Lancet.

[23] Kwok CS, Loke YK (2010). Meta-analysis: the effects of proton pump inhibitors on cardiovascular events and mortality in patients receiving clopidogrel. Aliment Pharmacol Ther.

[24] Deshpande G, Rao S, Patole S, Bulsara M (2010). Updated meta-analysis of probiotics for preventing necrotizing enterocolitis in preterm neonates. Pediatrics.

[25] Leonard CE (2018). Comparative Safety of Sulfonylureas and the Risk of Sudden Cardiac Arrest and Ventricular Arrhythmia. Diabetes Care.

[26] Siscovick DS (2010). Type 2 diabetes mellitus and the risk of sudden cardiac arrest in the community. Rev Endocr Metab Disord.

[27] Kucharska-Newton AM (2010). Diabetes and the risk of sudden cardiac death, the Atherosclerosis Risk in Communities study. Acta Diabetol.

[28] Nichol G (2008). Regional variation in out-of-hospital cardiac arrest incidence and outcome. JAMA.

[29] El-Atat FA, McFarlane SI, Sowers JR, Bigger JT (2004). Sudden cardiac death in patients with diabetes. Curr Diab Rep.

[30] Center for Medicare & Medicaid Services. Medicaid, https://www.medicaid.gov/medicaid/index.html. Last accessed: 10 February 2020.

[31] O’Malley KJ (2005). Measuring diagnoses: ICD code accuracy. Health Serv Res.

[32] Hennessy S (2010). Validation of diagnostic codes for outpatient-originating sudden cardiac death and ventricular arrhythmia in Medicaid and Medicare claims data. Pharmacoepidemiol Drug Saf.

[33] By the American Geriatrics Society Beers Criteria Update Expert, P. American Geriatrics Society 2015 Updated Beers Criteria for Potentially Inappropriate Medication Use in Older Adults. *Journal of the American Geriatrics Society* **63**, 2227–2246, 10.1111/jgs.13702 (2015). 10.1111/jgs.13702 26446832

[34] Bunch TJ (2004). Prediction of short- and long-term outcomes by electrocardiography in survivors of out-of-hospital cardiac arrest. Resuscitation.

[35] Manfredini R, Portaluppi F, Grandi E, Fersini C, Gallerani M (1996). Out-of-hospital sudden death referring to an emergency department. J Clin Epidemiol.

[36] Myerburg RJ, Kessler KM, Zaman L, Conde CA, Castellanos A (1982). Survivors of prehospital cardiac arrest. JAMA.

[37] Benjamin EJ (2019). Heart Disease and Stroke Statistics-2019 Update: A Report From the American Heart Association. Circulation.

[38] Liberthson RR (1996). Sudden death from cardiac causes in children and young adults. New England Journal of Medicine.

[39] Statista. Market share of leading health insurance companies in the United States in 2018, by direct premiums written, https://www.statista.com/statistics/216518/leading-us-health-insurance-groups-in-the-us/. Last accessed: 13 May 2020. (2019).

[40] Kitzmiller JL (2008). Managing preexisting diabetes for pregnancy: summary of evidence and consensus recommendations for care. Diabetes Care.

[41] CredibleMeds. Congenital LQTS and Drugs to Avoid List, https://www.crediblemeds.org/everyone/congenital-long-qt-and-drugs-avoid/. Last accessed: 10 February 2020.

[42] Suissa S (2008). Immeasurable time bias in observational studies of drug effects on mortality. Am J Epidemiol.

[43] Lund JL, Richardson DB, Sturmer T (2015). The active comparator, new user study design in pharmacoepidemiology: historical foundations and contemporary application. Curr Epidemiol Rep.

[44] Yoshida K, Solomon DH, Kim SC (2015). Active-comparator design and new-user design in observational studies. Nat Rev Rheumatol.

[45] Zunkler BJ (2006). Human ether-a-go-go-related (HERG) gene and ATP-sensitive potassium channels as targets for adverse drug effects. Pharmacol Ther.

[46] Pantalone, K. M. *et al*. Increase in overall mortality risk in patients with type 2 diabetes receiving glipizide, glyburide or glimepiride monotherapy versus metformin: a retrospective analysis. *Diabetes Obes Metab* **14**, 803–809, 10.1111/j.1463-1326.2012.01604.x (2012). 10.1111/j.1463-1326.2012.01604.x 22486923

[47] Law V (2014). DrugBank 4.0: shedding new light on drug metabolism. Nucleic Acids Res.

[48] Zunkler BJ, Lenzen S, Manner K, Panten U, Trube G (1988). Concentration-dependent effects of tolbutamide, meglitinide, glipizide, glibenclamide and diazoxide on ATP-regulated K+ currents in pancreatic B-cells. Naunyn Schmiedebergs Arch Pharmacol.

[49] Flynn DM (2005). The sulfonylurea glipizide does not inhibit ischemic preconditioning in anesthetized rabbits. Cardiovasc Drugs Ther.

[50] Toh S, Garcia Rodriguez LA, Hernan MA (2011). Confounding adjustment via a semi-automated high-dimensional propensity score algorithm: an application to electronic medical records. Pharmacoepidemiol Drug Saf.

[51] Schneeweiss S (2009). High-dimensional propensity score adjustment in studies of treatment effects using health care claims data. Epidemiology.

[52] Rassen, J. A., Doherty, M., Huang, W. & Schneeweiss, S. Pharmacoepidemiology Toolbox including High-dimensional Propensity Score (hd-PS), http://www.drugepi.org/dope-downloads/#Pharmacoepidemiology%20Toolbox. Last accessed: 10 February 2020. (2017).

[53] Rassen JA, Glynn RJ, Brookhart MA, Schneeweiss S (2011). Covariate selection in high-dimensional propensity score analyses of treatment effects in small samples. Am J Epidemiol.

[54] Austin PC (2008). Goodness-of-fit diagnostics for the propensity score model when estimating treatment effects using covariate adjustment with the propensity score. Pharmacoepidemiol Drug Saf.

