# Supplementary material for: Risk of sudden cardiac arrest and ventricular arrhythmia with sulfonylureas: An experience with conceptual replication in two independent populations
Source: Sci Rep. 2020 Jun 22;10:10070. doi: 10.1038/s41598-020-66668-5 (PMC7308403; doi:10.1038/s41598-020-66668-5)
Supplement: Supplementary file 1 — Supplementary information. [file 41598_2020_66668_MOESM1_ESM.docx]

**Supplementary Information**

Supplementary information for “Risk of sudden cardiac arrest and ventricular arrhythmia with sulfonylureas: An experience with conceptual replication in two independent populations” by Neil Dhopeshwarkar^1^, Colleen M. Brensinger^1^, Warren B. Bilker^1^, Samantha E. Soprano^1^, James H. Flory^1,2^, Ghadeer K. Dawwas^1^, Joshua J. Gagne^3^, Sean Hennessy^1,4^, and Charles E. Leonard^1*^

1. Center for Pharmacoepidemiology Research and Training, Department of Biostatistics, Epidemiology, and Informatics, Perelman School of Medicine, University of Pennsylvania (Philadelphia, Pennsylvania)
2. Endocrinology Service, Department of Subspecialty Medicine, Memorial Sloan Kettering Cancer Center (New York, New York)
3. Division of Pharmacoepidemiology and Pharmacoeconomics, Department of Medicine, Brigham and Women’s Hospital and Harvard Medical School (Boston, Massachusetts)
4. Department of Systems Pharmacology and Translational Therapeutics, Perelman School of Medicine, University of Pennsylvania (Philadelphia, Pennsylvania)

*Corresponding author

**Supplementary Table 1. Characteristics of individual second-generation sulfonylurea users in Medicaid**

|  | | **Glipizide** | **Glimepiride** | | **Glyburide** | |
| --- | --- | --- | --- | --- | --- | --- |
| Users, N | | 268,094 | 124,354 | | 231,958 | |
| Person-years of follow-up, sum, among all users | | 86,335 | 40,473 | | 74,374 | |
| Days of follow-up, median (5^th^, 95^th^ percentile), per user | | 46 (1, 434) | 52 (1, 429) | | 46 (1, 422) | |
| Proportion of follow-up time covered by days' supply of given sulfonylurea dispensings, median (5^th^, 95^th^ percentile) | | 87.4 (67.4, 100.0) | 89.6 (67.4, 100.0) | | 86.0 (67.4, 100.0) | |
| **Demographics** | Group | % (unless otherwise noted) | % (unless otherwise noted) | WCSD^ǁ^ | % (unless otherwise noted) | WCSD^ǁ^ |
| Age, in years, at cohort entry | Median (Q1-Q3) | 57.6 (48.0-66.9) | 58.6 (49.0-67.4) | 0.04 | 57.7 (47.8-67.1) | 0.06 |
| Sex | Female | 59.7 | 61.0 | 0.01 | 59.3 | 0.01 |
| Race/Ethnicity | White | 33.6 | 41.6 | 0.07 | 32.0 | 0.01 |
|  | Black | 20.7 | 15.1 | 0.04 | 18.4 | 0.03 |
|  | Hispanic/Latino | 24.2 | 19.1 | 0.06 | 25.5 | 0.02 |
|  | Asian | 5.5 | 7.8 | 0.07 | 6.9 | 0.02 |
|  | Unknown/Other | 16.0 | 16.4 | 0.04 | 17.2 | 0.01 |
| State of residence | CA | 43.2 | 41.1 | 0.07 | 49.1 | 0.09 |
|  | FL | 13.2 | 10.6 | 0.08 | 11.2 | 0.07 |
|  | NY | 27.2 | 27.4 | 0.03 | 26.8 | 0.05 |
|  | OH | 7.8 | 13.3 | 0.10 | 7.5 | 0.05 |
|  | PA | 8.6 | 7.7 | 0.05 | 5.4 | 0.06 |
| Census level | New England | 0.0 | 0.0 | 0.00 | 0.0 | 0.00 |
|  | Middle Atlantic | 35.8 | 35.0 | 0.05 | 32.2 | 0.03 |
|  | East North Central | 7.8 | 13.3 | 0.10 | 7.5 | 0.05 |
|  | West North Central | 0.0 | 0.0 | 0.00 | 0.0 | 0.00 |
|  | South Atlantic | 13.2 | 10.6 | 0.08 | 11.2 | 0.07 |
|  | East South Central | 0.0 | 0.0 | 0.00 | 0.0 | 0.00 |
|  | West South Central | 0.0 | 0.0 | 0.00 | 0.0 | 0.00 |
|  | Mountain | 0.0 | 0.0 | 0.00 | 0.0 | 0.00 |
|  | Pacific | 43.2 | 41.1 | 0.07 | 49.1 | 0.09 |
|  | Unknown | 0.0 | 0.0 | 0.00 | 0.0 | 0.00 |
| Calendar year of cohort entry^‡^ | 2000 | 6.5 | 5.0 | 0.02 | 6.6 | 0.04 |
|  | 2001 | 7.2 | 4.7 | 0.06 | 9.5 | 0.06 |
|  | 2002 | 7.4 | 5.6 | 0.04 | 9.5 | 0.04 |
|  | 2003 | 7.3 | 6.3 | 0.03 | 8.8 | 0.02 |
|  | 2004 | 5.9 | 5.9 | 0.02 | 7.1 | 0.02 |
|  | 2005 | 7.8 | 7.2 | 0.03 | 8.9 | 0.02 |
|  | 2006 | 11.5 | 10.1 | 0.05 | 11.5 | 0.02 |
|  | 2007 | 7.6 | 8.3 | 0.02 | 7.3 | 0.02 |
|  | 2008 | 6.3 | 7.2 | 0.03 | 5.9 | 0.02 |
|  | 2009 | 7.2 | 8.4 | 0.03 | 6.2 | 0.02 |
|  | 2010 | 8.4 | 10.5 | 0.06 | 7.2 | 0.03 |
|  | 2011 | 7.9 | 9.6 | 0.05 | 5.8 | 0.04 |
|  | 2012 | 9.2 | 11.2 | 0.06 | 5.7 | 0.09 |
| Medicare enrolled | Yes | 49.1 | 51.4 | 0.03 | 46.8 | 0.05 |
| Nursing home residence ever during baseline | Yes | 7.0 | 5.6 | 0.03 | 5.1 | 0.02 |
| **Healthcare use intensity measures, in baseline period^*^** | Group | Measure of central tendency | Measure of central tendency | WCSD | Measure of central tendency | WCSD |
| No. prescriptions dispensed, total | Median (Q1-Q3) | 36.0 (8.0-77.0) | 50.0 (18.0-92.0) | 0.05 | 31.0 (6.0-71.0) | 0.02 |
| No. prescriptions dispensed, by unique drug | Median (Q1-Q3) | 11.0 (5.0-18.0) | 14.0 (7.0-21.0) | 0.08 | 10.0 (4.0-18.0) | 0.05 |
| No. outpatient diagnosis codes, total | Median (Q1-Q3) | 28.0 (9.0-71.0) | 35.0 (14.0-80.0) | 0.05 | 26.0 (9.0-62.0) | 0.06 |
| No. outpatient diagnosis codes, by unique code | Median (Q1-Q3) | 11.0 (5.0-21.0) | 13.0 (6.0-24.0) | 0.06 | 11.0 (5.0-20.0) | 0.10 |
| No. outpatient CPT-4/HCPCS codes, total | Median (Q1-Q3) | 34.0 (11.0-79.0) | 43.0 (17.0-91.0) | 0.07 | 31.0 (10.0-70.0) | 0.11 |
| No. outpatient CPT-4/HCPCS codes, by unique code | Median (Q1-Q3) | 21.0 (8.0-40.0) | 25.0 (11.0-45.0) | 0.06 | 20.0 (8.0-37.0) | 0.10 |
| **Other investigator pre-defined covariates, in baseline period** | Group | % | % | WCSD | % | WCSD |
| Disorders of lipid metabolism | Yes | 41.0 | 50.9 | 0.08 | 39.6 | 0.05 |
| Rheumatic heart disease, chronic | Yes | 2.4 | 2.5 | 0.01 | 2.1 | 0.02 |
| Hypertensive disease | Yes | 57.6 | 62.7 | 0.06 | 55.8 | 0.06 |
| Ischemic heart disease | Yes | 20.8 | 23.7 | 0.05 | 19.5 | 0.05 |
| Conduction disorders | Yes | 2.1 | 2.1 | 0.01 | 1.8 | 0.01 |
| Heart failure/cardiomyopathy | Yes | 13.4 | 13.6 | 0.03 | 11.6 | 0.03 |
| Cardiomegaly | Yes | 6.2 | 6.2 | 0.02 | 5.2 | 0.02 |
| Congenital anomalies of the heart, other | Yes | 1.3 | 1.4 | 0.02 | 1.3 | 0.02 |
| Implantable cardioverter defibrillator/pacemaker use | Yes | 1.0 | 1.1 | 0.01 | 0.8 | 0.00 |
| Kidney disease | Yes | 17.5 | 18.6 | 0.04 | 13.5 | 0.03 |
| Depression | Yes | 24.6 | 28.3 | 0.04 | 22.7 | 0.01 |
| Obesity | Yes | 12.0 | 13.4 | 0.02 | 10.5 | 0.01 |
| Tobacco use | Yes | 9.1 | 9.5 | 0.01 | 7.6 | 0.02 |
| Alcohol abuse | Yes | 3.5 | 2.6 | 0.02 | 3.3 | 0.01 |
| Hypoglycemia, serious | Yes | 2.3 | 2.2 | 0.01 | 2.2 | 0.01 |
| Diabetes mellitus, type 2^†^ | Yes | 95.2 | 94.8 | 0.02 | 95.1 | 0.01 |
| Adapted Diabetes Complications Severity Index | 0 | 50.7 | 46.1 | 0.05 | 53.2 | 0.07 |
|  | 1 | 12.0 | 13.8 | 0.03 | 12.4 | 0.00 |
|  | 2 | 14.5 | 14.9 | 0.01 | 14.5 | 0.01 |
|  | 3 | 6.8 | 7.8 | 0.02 | 6.4 | 0.02 |
|  | 4 | 5.9 | 6.3 | 0.02 | 5.4 | 0.02 |
|  | 5+ | 10.0 | 11.1 | 0.02 | 8.1 | 0.02 |
| **Drugs in the 30 days prior to cohort entry^**^** | Group | % | % | WCSD | % | WCSD |
| alpha-glucosidase inhibitor | Yes | 0.2 | 0.4 | 0.01 | 0.2 | 0.00 |
| amylin analog | Yes | 0.0 | 0.0 | 0.01 | 0.0 | 0.00 |
| dipeptidyl peptidase-4 inhibitor | Yes | 1.6 | 3.9 | 0.04 | 1.1 | 0.01 |
| glucagon-like peptide-1 receptor agonist | Yes | 0.2 | 0.6 | 0.02 | 0.1 | 0.00 |
| insulin | Yes | 7.6 | 10.1 | 0.04 | 6.7 | 0.02 |
| metformin | Yes | 22.4 | 27.4 | 0.06 | 19.5 | 0.06 |
| meglitinide | Yes | 0.7 | 1.7 | 0.03 | 0.8 | 0.01 |
| sodium-glucose co-transporter 2 inhibitor§ | Yes | 0.0 | 0.0 | 0.00 | 0.0 | 0.00 |
| thiazolidinedione | Yes | 7.1 | 11.6 | 0.08 | 6.5 | 0.01 |
| CYP2C9 inhibitor | Yes | 4.9 | 4.8 | 0.01 | 4.7 | 0.01 |
| CYP3A4 inhibitor | Yes | 3.7 | 3.8 | 0.01 | 3.5 | 0.00 |
| CYP2C9 inducer | Yes | 0.9 | 0.9 | 0.01 | 0.9 | 0.00 |
| CYP3A4 inducer | Yes | 5.8 | 8.6 | 0.04 | 5.3 | 0.01 |
| drug with known risk of TdP^§§^ | Yes | 9.1 | 10.5 | 0.02 | 8.1 | 0.01 |
| drug with known, possible, or conditional risk of TdP^§§^ | Yes | 45.1 | 50.6 | 0.05 | 41.7 | 0.00 |
| ≥ 5 prescription dispensings for unique drugs in 30 days prior to entry | Yes | 39.7 | 47.7 | 0.05 | 36.1 | 0.01 |
| CA = California; CPT = Current Procedural Terminology; CYP = hepatic cytochrome P450; FL = Florida; HCPCS = Healthcare Common Procedure Coding System; NY = New York; OH = Ohio; PA = Pennsylvania; Q = quartile; TdP = torsade de pointes; WCSD = weighted conditional standardized difference  * The following healthcare utilization covariates were excluded from presentation in the table, as their median values were zero for each sulfonylurea: # inpatient ICD-9 diagnosis codes; # unique inpatient ICD-9 diagnosis codes; # inpatient ICD-9 procedure codes; # unique inpatient ICD-9 procedure codes; # inpatient CPT/HCPCS procedure codes; # unique inpatient CPT/HCPCS procedure codes; # outpatient ICD-9 procedure codes; # unique outpatient ICD-9 procedure codes; # other setting ICD-9 diagnosis codes; # unique other setting ICD-9 diagnosis codes; # other setting ICD-9 procedure codes; # unique other setting ICD-9 procedure codes  ** Antimicrobial drugs in each category were examined within 14 (rather than 30) days prior to cohort entry; these agents are typically prescribed for acute conditions  † Defined by ratio of type 1 (ICD-9 250.X1 or 250.X3) to type 2 (ICD-9 250.X0 or 250.X2) codes ≤0.5, ascertained during baseline and on cohort entry date  ‡ Prespecified covariate not forced into propensity score, but included as a categorical variable in outcome model  § Not marketed during years of study  §§ Per CredibleMeds (AZCERT Inc.: Oro Valley, AZ)  ǁ Vs. glipizide | | | | | | |

**Supplementary Table 2. Characteristics of individual second-generation sulfonylurea users in Optum**

|  | | **Glipizide** | **Glimepiride** | | **Glyburide** | |
| --- | --- | --- | --- | --- | --- | --- |
| Users, N | | 206,034 | 151,229 | | 134,677 | |
| Person-years of follow-up, sum, among all users | | 85,565 | 59,246 | | 53,037 | |
| Days of follow-up, median (5^th^, 95^th^ percentile), per user | | 77 (2, 551) | 76 (3, 507) | | 72 (4, 533) | |
| Proportion of follow-up time covered by days' supply of given sulfonylurea dispensings, median (5^th^, 95^th^ percentile) | | 89.1 (67.4, 100.0) | 88.9 (67.4, 100.0) | | 85.8 (67.4, 100.0) | |
| **Demographics** | Group | % (unless otherwise noted) | % (unless otherwise noted) | WCSD^§§^ | % (unless otherwise noted) | WCSD^§§^ |
| Age, in years, at cohort entry | Median (Q1-Q3) | 58.8 (50.2-67.3) | 58.1 (49.9-66.4) | 0.10 | 57.4 (49.0-66.3) | 0.07 |
| Sex | Female | 44.2 | 44.3 | 0.02 | 43.2 | 0.02 |
| Race/Ethnicity | White | 55.8 | 61.3 | 0.03 | 53.6 | 0.05 |
|  | Black | 13.7 | 13.6 | 0.03 | 12.3 | 0.04 |
|  | Hispanic/Latino | 16.3 | 12.4 | 0.05 | 17.3 | 0.02 |
|  | Asian | 3.7 | 3.6 | 0.01 | 3.8 | 0.02 |
|  | Unknown/Missing | 10.5 | 9.1 | 0.03 | 13.0 | 0.05 |
| Census level | New England | 2.9 | 2.0 | 0.01 | 2.8 | 0.01 |
|  | Middle Atlantic | 4.8 | 5.8 | 0.02 | 3.7 | 0.00 |
|  | East North Central | 12.4 | 15.2 | 0.08 | 11.9 | 0.03 |
|  | West North Central | 8.1 | 8.8 | 0.05 | 7.1 | 0.06 |
|  | South Atlantic | 27.5 | 30.4 | 0.07 | 24.7 | 0.06 |
|  | East South Central | 3.9 | 6.2 | 0.04 | 4.4 | 0.05 |
|  | West South Central | 15.8 | 16.6 | 0.04 | 17.3 | 0.07 |
|  | Mountain | 7.4 | 5.5 | 0.03 | 7.3 | 0.02 |
|  | Pacific | 16.5 | 9.1 | 0.07 | 20.0 | 0.12 |
|  | Unknown | 0.7 | 0.4 | 0.01 | 0.7 | 0.02 |
| Education level | Less than 12th Grade | 2.0 | 1.2 | 0.02 | 2.5 | 0.01 |
|  | High School Diploma | 37.9 | 37.5 | 0.02 | 36.6 | 0.03 |
|  | Less than Bachelor Degree | 44.8 | 46.4 | 0.01 | 43.3 | 0.03 |
|  | Bachelor Degree Plus | 7.4 | 8.4 | 0.01 | 7.3 | 0.01 |
|  | Unknown | 8.0 | 6.5 | 0.04 | 10.3 | 0.05 |
| Housing | Probable Homeowner | 68.0 | 72.2 | 0.01 | 67.3 | 0.03 |
|  | Unknown | 32.0 | 27.8 | 0.01 | 32.7 | 0.03 |
| Household income | <$40K | 19.4 | 17.7 | 0.01 | 16.8 | 0.04 |
|  | $40K-$49K | 7.1 | 7.2 | 0.01 | 6.3 | 0.03 |
|  | $50K-$59K | 6.7 | 7.0 | 0.02 | 6.0 | 0.02 |
|  | $60K-$74K | 8.6 | 9.4 | 0.01 | 7.5 | 0.03 |
|  | $75K-$99K | 10.7 | 12.2 | 0.02 | 9.5 | 0.03 |
|  | $100K+ | 15.8 | 20.4 | 0.02 | 14.5 | 0.03 |
|  | Unknown | 31.7 | 26.2 | 0.01 | 39.4 | 0.06 |
| Total net worth of the primary customer | <$25K | 12.0 | 10.7 | 0.01 | 10.9 | 0.03 |
|  | $25K-$149K | 21.7 | 21.4 | 0.01 | 20.6 | 0.02 |
|  | $150K-$249K | 14.3 | 15.2 | 0.01 | 14.1 | 0.01 |
|  | $250K-$499K | 21.1 | 22.7 | 0.01 | 21.0 | 0.02 |
|  | $500K+ | 13.8 | 15.2 | 0.02 | 14.9 | 0.02 |
|  | Unknown | 17.1 | 14.7 | 0.01 | 18.6 | 0.04 |
| Calendar year of cohort entry^‡^ | 2001 | 2.9 | 1.8 | 0.04 | 5.7 | 0.10 |
|  | 2002 | 3.7 | 3.1 | 0.04 | 6.1 | 0.07 |
|  | 2003 | 3.8 | 3.0 | 0.04 | 5.2 | 0.05 |
|  | 2004 | 3.9 | 3.4 | 0.04 | 5.3 | 0.05 |
|  | 2005 | 12.8 | 6.2 | 0.11 | 19.7 | 0.07 |
|  | 2006 | 6.4 | 6.4 | 0.05 | 8.2 | 0.07 |
|  | 2007 | 5.7 | 5.8 | 0.04 | 7.3 | 0.08 |
|  | 2008 | 6.8 | 7.3 | 0.04 | 8.2 | 0.10 |
|  | 2009 | 5.9 | 6.9 | 0.04 | 6.6 | 0.10 |
|  | 2010 | 6.2 | 7.3 | 0.04 | 6.5 | 0.11 |
|  | 2011 | 6.6 | 7.8 | 0.04 | 6.2 | 0.10 |
|  | 2012 | 6.7 | 8.2 | 0.03 | 5.5 | 0.11 |
|  | 2013 | 6.8 | 8.5 | 0.03 | 4.0 | 0.09 |
|  | 2014 | 6.6 | 7.5 | 0.02 | 2.0 | 0.14 |
|  | 2015 | 7.3 | 8.1 | 0.02 | 1.8 | 0.15 |
|  | 2016 | 7.9 | 8.7 | 0.04 | 1.8 | 0.08 |
| Medicare enrolled | Yes | 34.1 | 28.7 | 0.13 | 27.1 | 0.12 |
| Nursing home residence ever during baseline | Yes | 1.6 | 1.1 | 0.02 | 1.1 | 0.01 |
| **Healthcare use intensity measures, in baseline period^*^** | Group | Measure of central tendency | Measure of central tendency | WCSD | Measure of central tendency | WCSD |
| No. prescriptions dispensed, total | Median (Q1-Q3) | 18.0 (4.0-41.0) | 25.0 (9.0-47.0) | 0.05 | 12.0 (2.0-33.0) | 0.04 |
| No. prescriptions dispensed, by unique drug | Median (Q1-Q3) | 6.0 (3.0-11.0) | 8.0 (4.0-12.0) | 0.05 | 5.0 (2.0-10.0) | 0.06 |
| No. outpatient diagnosis codes, total | Median (Q1-Q3) | 19.0 (6.0-40.0) | 23.0 (11.0-45.0) | 0.03 | 14.0 (3.0-32.0) | 0.07 |
| No. outpatient diagnosis codes, by unique code | Median (Q1-Q3) | 10.0 (4.0-17.0) | 11.0 (6.0-18.0) | 0.05 | 8.0 (2.0-14.0) | 0.09 |
| No. outpatient CPT-4/HCPCS codes, total | Median (Q1-Q3) | 21.0 (7.0-43.0) | 26.0 (12.0-49.0) | 0.03 | 16.0 (3.0-34.0) | 0.07 |
| No. outpatient CPT-4/HCPCS codes, by unique code | Median (Q1-Q3) | 14.0 (5.0-26.0) | 17.0 (9.0-29.0) | 0.05 | 11.0 (2.0-22.0) | 0.08 |
| **Other investigator pre-defined covariates, in baseline period** | Group | % | % | WCSD | % | WCSD |
| Disorders of lipid metabolism | Yes | 55.4 | 66.0 | 0.07 | 48.1 | 0.06 |
| Rheumatic heart disease, chronic | Yes | 1.2 | 1.3 | 0.01 | 1.0 | 0.01 |
| Hypertensive disease | Yes | 60.5 | 67.8 | 0.07 | 52.3 | 0.06 |
| Ischemic heart disease | Yes | 14.6 | 15.6 | 0.02 | 12.4 | 0.03 |
| Conduction disorders | Yes | 1.7 | 1.7 | 0.00 | 1.2 | 0.01 |
| Heart failure/cardiomyopathy | Yes | 6.5 | 6.0 | 0.01 | 4.8 | 0.03 |
| Cardiomegaly | Yes | 3.2 | 3.1 | 0.02 | 2.4 | 0.02 |
| Congenital anomalies of the heart, other | Yes | 0.4 | 0.4 | 0.01 | 0.4 | 0.01 |
| Implantable cardioverter defibrillator/pacemaker use | Yes | 0.9 | 0.9 | 0.00 | 0.6 | 0.00 |
| Kidney disease | Yes | 14.8 | 15.4 | 0.02 | 9.6 | 0.05 |
| Depression | Yes | 14.2 | 16.5 | 0.03 | 10.7 | 0.03 |
| Obesity | Yes | 14.7 | 16.3 | 0.02 | 10.6 | 0.03 |
| Tobacco use | Yes | 9.7 | 9.7 | 0.01 | 6.7 | 0.03 |
| Alcohol abuse | Yes | 1.5 | 1.2 | 0.00 | 1.0 | 0.03 |
| Hypoglycemia, serious | Yes | 0.7 | 0.5 | 0.01 | 0.5 | 0.01 |
| Diabetes mellitus, type 2^†^ | Yes | 98.1 | 97.9 | 0.01 | 97.8 | 0.02 |
| Adapted Diabetes Complications Severity Index | 0 | 60.3 | 56.9 | 0.03 | 67.2 | 0.05 |
|  | 1 | 13.8 | 16.0 | 0.04 | 12.4 | 0.03 |
|  | 2 | 12.2 | 13.0 | 0.02 | 10.5 | 0.03 |
|  | 3 | 5.3 | 5.8 | 0.01 | 4.1 | 0.01 |
|  | 4 | 3.8 | 3.8 | 0.01 | 2.8 | 0.02 |
|  | 5+ | 4.7 | 4.5 | 0.01 | 2.9 | 0.01 |
| **Drugs in the 30 days prior to cohort entry^**^** | Group | % | % | WCSD | % | WCSD |
| alpha-glucosidase inhibitor | Yes | 0.1 | 0.1 | 0.00 | 0.1 | 0.01 |
| amylin analog | Yes | 0.0 | 0.0 | 0.01 | 0.0 | 0.00 |
| dipeptidyl peptidase-4 inhibitor | Yes | 3.2 | 5.7 | 0.02 | 1.8 | 0.04 |
| glucagon-like peptide-1 receptor agonist | Yes | 0.8 | 1.7 | 0.02 | 0.5 | 0.01 |
| insulin | Yes | 3.8 | 4.6 | 0.02 | 2.8 | 0.01 |
| metformin | Yes | 25.0 | 28.6 | 0.02 | 18.8 | 0.13 |
| meglitinide | Yes | 0.3 | 0.6 | 0.02 | 0.3 | 0.01 |
| sodium-glucose co-transporter 2 inhibitor | Yes | 0.3 | 0.6 | 0.01 | 0.1 | 0.02 |
| thiazolidinedione | Yes | 5.0 | 6.3 | 0.03 | 5.3 | 0.04 |
| CYP2C9 inhibitor | Yes | 2.8 | 2.7 | 0.00 | 2.6 | 0.01 |
| CYP3A4 inhibitor | Yes | 2.1 | 2.1 | 0.00 | 2.0 | 0.00 |
| CYP2C9 inducer | Yes | 0.2 | 0.2 | 0.00 | 0.2 | 0.01 |
| CYP3A4 inducer | Yes | 3.4 | 4.5 | 0.02 | 3.3 | 0.03 |
| drug with known risk of TdP^§^ | Yes | 6.3 | 6.9 | 0.01 | 5.4 | 0.01 |
| drug with known, possible, or conditional risk of TdP^§^ | Yes | 32.5 | 34.9 | 0.02 | 28.3 | 0.03 |
| ≥ 5 prescription dispensings for unique drugs in 30 days prior to entry | Yes | 21.7 | 24.9 | 0.03 | 18.1 | 0.04 |
| **Laboratory covariates, in baseline period** | Group | % | % | WCSD | % | WCSD |
| Blood glucose | No lab ever in past | 51.6 | 42.9 | 0.05 | 59.8 | 0.08 |
|  | No lab in 1-year baseline | 22.8 | 26.5 | 0.05 | 20.9 | 0.06 |
|  | Normal | 8.4 | 10.0 | 0.02 | 6.0 | 0.02 |
|  | Low abnormal | 0.2 | 0.2 | 0.00 | 0.2 | 0.01 |
|  | High abnormal | 16.9 | 20.4 | 0.04 | 13.1 | 0.08 |
| Hemoglobin A1c | No lab ever in past | 51.6 | 42.9 | 0.05 | 59.8 | 0.08 |
|  | No lab in 1-year baseline | 25.2 | 29.3 | 0.05 | 23.4 | 0.06 |
|  | Normal | 6.1 | 7.2 | 0.02 | 4.4 | 0.03 |
|  | Low abnormal | 0.0 | 0.0 | 0.00 | 0.0 | 0.00 |
|  | High abnormal | 17.0 | 20.6 | 0.04 | 12.4 | 0.06 |
| Serum creatinine | No lab ever in past | 51.6 | 42.9 | 0.05 | 59.8 | 0.08 |
|  | No lab in 1-year baseline | 22.6 | 26.2 | 0.06 | 21.0 | 0.06 |
|  | Normal | 19.2 | 23.5 | 0.05 | 15.5 | 0.07 |
|  | Low abnormal | 3.6 | 4.3 | 0.01 | 2.2 | 0.02 |
|  | High abnormal | 2.9 | 3.0 | 0.01 | 1.5 | 0.02 |
| Hematocrit | No lab ever in past | 51.6 | 42.9 | 0.05 | 59.8 | 0.08 |
|  | No lab in 1-year baseline | 29.5 | 35.3 | 0.05 | 26.4 | 0.07 |
|  | Normal | 16.5 | 19.3 | 0.04 | 12.3 | 0.05 |
|  | Low abnormal | 2.1 | 2.2 | 0.01 | 1.3 | 0.01 |
|  | High abnormal | 0.3 | 0.3 | 0.00 | 0.2 | 0.00 |
| Hemoglobin | No lab ever in past | 51.6 | 42.9 | 0.05 | 59.8 | 0.08 |
|  | No lab in 1-year baseline | 29.5 | 35.4 | 0.05 | 26.4 | 0.06 |
|  | Normal | 16.0 | 18.7 | 0.04 | 12.0 | 0.05 |
|  | Low abnormal | 2.4 | 2.5 | 0.01 | 1.4 | 0.01 |
|  | High abnormal | 0.4 | 0.4 | 0.00 | 0.4 | 0.01 |
| Hypoglycemia, laboratory measured, alert value | No lab ever in past | 51.6 | 42.9 | 0.05 | 59.8 | 0.08 |
|  | No lab in 1-year baseline | 22.8 | 26.5 | 0.05 | 20.9 | 0.06 |
|  | No alert value | 25.0 | 30.0 | 0.04 | 18.9 | 0.07 |
|  | Alert value | 0.5 | 0.5 | 0.01 | 0.4 | 0.02 |
| Hypoglycemia, laboratory measured, clinically significant value | No lab ever in past | 51.6 | 42.9 | 0.05 | 59.8 | 0.08 |
|  | No glucose in 1-year baseline | 22.8 | 26.5 | 0.05 | 20.9 | 0.06 |
|  | No clinically significant value | 25.3 | 30.4 | 0.04 | 19.2 | 0.07 |
|  | Clinically significant value | 0.2 | 0.2 | 0.00 | 0.1 | 0.00 |
| CPT = Current Procedural Terminology; CYP = hepatic cytochrome P450; HCPCS = Healthcare Common Procedure Coding System; Q = quartile; TdP = torsade de pointes; WCSD = weighted conditional standardized difference  * The following healthcare utilization covariates were excluded from presentation in the table, as their median values were zero for each sulfonylurea: # inpatient ICD-9 diagnosis codes; # unique inpatient ICD-9 diagnosis codes; # inpatient ICD-9 procedure codes; # unique inpatient ICD-9 procedure codes; # inpatient CPT/HCPCS procedure codes; # unique inpatient CPT/HCPCS procedure codes; # outpatient ICD-9 procedure codes; # unique outpatient ICD-9 procedure codes; # other setting ICD-9 diagnosis codes; # unique other setting ICD-9 diagnosis codes; # other setting ICD-9 procedure codes; # unique other setting ICD-9 procedure codes; # laboratory LOINC codes; # unique laboratory LOINC codes  ** Antimicrobial drugs in each category were examined within 14 (rather than 30) days prior to cohort entry; these agents are typically prescribed for acute conditions  † Defined by ratio of type 1 (ICD-9 250.X1 or 250.X3) to type 2 (ICD-9 250.X0 or 250.X2) codes ≤0.5, ascertained during baseline and on cohort entry date  ‡ Prespecified covariate not forced into propensity score, but included as a categorical variable in outcome model  § Per CredibleMeds (AZCERT Inc.: Oro Valley, AZ)  §§ Vs. glipizide | | | | | | |

**Supplementary Table 3. Pre-specified covariates included in propensity score**

| **Category** | **Covariate** |
| --- | --- |
| Demographic | age at cohort entry (deciles) |
|  | sex |
|  | race |
|  | state of residence* |
|  | census level division based on US state** |
|  | enrollment in Medicare |
|  | nursing home residence ever during baseline |
| Socioeconomic status** | education level |
|  | home ownership |
|  | household income range |
|  | total net worth of the primary customer |
| Healthcare utilization intensity | number of prescriptions dispensed |
|  | number of unique drugs dispensed |
|  | number of inpatient ICD-9/ICD-10 diagnoses |
|  | number of unique inpatient ICD-9/ICD-10 diagnoses |
|  | number of inpatient ICD-9/ICD-10 procedure codes |
|  | number of unique inpatient ICD-9/ICD-10 procedure codes |
|  | number of inpatient CPT-4/HCPCS procedure codes |
|  | number of unique inpatient CPT-4/HCPCS procedure codes |
|  | number of outpatient ICD-9/ICD-10 diagnoses |
|  | number of unique outpatient ICD-9/ICD-10 diagnoses |
|  | number of outpatient ICD-9/ICD-10 procedure codes |
|  | number of unique outpatient ICD-9/ICD-10 procedure codes |
|  | number of outpatient CPT-4/HCPCS procedure codes |
|  | number of unique outpatient CPT-4/HCPCS procedure codes |
|  | number of other setting ICD-9/ICD-10 diagnoses |
|  | number of unique other setting ICD-9/ICD-10 diagnoses |
|  | number of other setting ICD-9/ICD-10 procedure codes |
|  | number of unique other setting ICD-9/ICD-10 procedure codes |
|  | number of other setting CPT-4/HCPCS procedure codes |
|  | number of unique other setting CPT-4/HCPCS procedure codes |
|  | number of LOINC lab codes** |
|  | number of unique LOINC lab codes** |
| Comorbidities | adapted Diabetes Complications Severity Index^1^, as continuous measure^2^ |
|  | Alcohol abuse |
|  | Cardiomegaly |
|  | Conduction disorders |
|  | Congenital anomalies of the heart (other) |
|  | Depression |
|  | Disorders of lipid metabolism |
|  | Chronic rheumatic heart disease |
|  | Ischemic heart disease |
|  | Heart failure / cardiomyopathy |
|  | Hypertensive disease |
|  | Implantable cardioverter-defibrillator (ICD) / pacemaker use |
|  | Kidney disease |
|  | Obesity |
|  | Tobacco use |
|  | Prior serious hypoglycemia |
| Concomitant drug use | Polypharmacy |
|  | Alpha Glucosidase inhibitor |
|  | Amylin analog |
|  | Dipeptidyl peptidase-4 inhibitor |
|  | Glucagonlike peptide 1 receptor agonist |
|  | Insulin |
|  | Meglitinide |
|  | Metformin |
|  | Sodium-glucose cotransporter 2 inhibitor** |
|  | Thiazolidinedione |
|  | CYP2C9 inhibitors^3^ |
|  | CYP3A4 inhibitors^3^ |
|  | CYP2C9 inducers^3^ |
|  | CYP3A4 inducers^3^ |
|  | Drugs with any risk of TdP^4^ |
|  | Drugs with known risk of TdP^4^ |
| Laboratory** | creatinine |
|  | glucose |
|  | hematocrit |
|  | hemoglobin A1c |
|  | hemoglobin |
|  | hypoglycemia, alert value^5^ defined by blood glucose ≤10-70mg/dL |
|  | hypoglycemia, clinically significant value^5^ defined by blood glucose <10-54mg/dL |
| CYP = cytochrome P450; dL = deciliter; mg = milligrams; TdP = torsade de pointes  * Only included in Medicaid analysis  **Only included in Optum analysis  1. Glasheen et al. Diabetes Complications Severity Index (DCSI)-Update and ICD-10 translation. *J Diabetes Complications* 2017;31(6):1007-1013.  2. Young et al. Diabetes complications severity index and risk of mortality, hospitalization, and healthcare utilization. *Am J Manag Care* 2008;14(1):15-23.  3. Flockhart et al. The Flockhart Table™. Drug Interactions: Cytochrome P450 drug interaction table. Indiana University School of Medicine. 2019.  4. Woosley et al. CredibleMeds.org: What does it offer? *Trends Cardiovasc Med* 2018;28(2):94-99.  5. International Hypoglycaemia Study Group. Glucose concentrations of less than 3.0 mmol/L (54 mg/dL) should be reported in clinical trials: A joint position statement of the American Diabetes Association and the European Association for the Study of Diabetes. *Diabetes Care* 2017;40(1):155-157. | |

**Supplementary Table 4. Empirically identified covariates included in propensity score (Medicaid)**

| **Data Dimension** | **Code** | **Code description** | **Z-bias = 5*** |
| --- | --- | --- | --- |
| Drug | Drug-specific NDCs | acetaminophen |  |
| Drug | Drug-specific NDCs | albuterol |  |
| Drug | Drug-specific NDCs | alendronate (as alendronate sodium) |  |
| Drug | Drug-specific NDCs | allopurinol |  |
| Drug | Drug-specific NDCs | alprazolam |  |
| Drug | Drug-specific NDCs | amlodipine besylate | Y |
| Drug | Drug-specific NDCs | aripiprazole |  |
| Drug | Drug-specific NDCs | aspirin |  |
| Drug | Drug-specific NDCs | atenolol |  |
| Drug | Drug-specific NDCs | atorvastatin |  |
| Drug | Drug-specific NDCs | baclofen |  |
| Drug | Drug-specific NDCs | benazepril hydrochloride |  |
| Drug | Drug-specific NDCs | carvedilol |  |
| Drug | Drug-specific NDCs | citalopram (as citalopram hydrobromide) |  |
| Drug | Drug-specific NDCs | clonidine hydrochloride |  |
| Drug | Drug-specific NDCs | clopidogrel bisulfate |  |
| Drug | Drug-specific NDCs | codeine |  |
| Drug | Drug-specific NDCs | codeine phosphate | Y |
| Drug | Drug-specific NDCs | cyclobenzaprine hydrochloride | Y |
| Drug | Drug-specific NDCs | dextromethorphan hydrobromide |  |
| Drug | Drug-specific NDCs | diclofenac sodium |  |
| Drug | Drug-specific NDCs | digoxin |  |
| Drug | Drug-specific NDCs | escitalopram oxalate |  |
| Drug | Drug-specific NDCs | ezetimibe |  |
| Drug | Drug-specific NDCs | fenofibrate |  |
| Drug | Drug-specific NDCs | fexofenadine hydrochloride |  |
| Drug | Drug-specific NDCs | folic acid |  |
| Drug | Drug-specific NDCs | furosemide |  |
| Drug | Drug-specific NDCs | gabapentin | Y |
| Drug | Drug-specific NDCs | hydrochlorothiazide | Y |
| Drug | Drug-specific NDCs | hydrocodone bitartrate |  |
| Drug | Drug-specific NDCs | hydrocortisone |  |
| Drug | Drug-specific NDCs | hydroxyzine pamoate |  |
| Drug | Drug-specific NDCs | insulin aspart |  |
| Drug | Drug-specific NDCs | insulin glargine | Y |
| Drug | Drug-specific NDCs | insulin lispro |  |
| Drug | Drug-specific NDCs | insulin nph human recombinant |  |
| Drug | Drug-specific NDCs | insulin regular human recombinant |  |
| Drug | Drug-specific NDCs | ipratropium bromide |  |
| Drug | Drug-specific NDCs | isopropyl alcohol |  |
| Drug | Drug-specific NDCs | isosorbide mononitrate |  |
| Drug | Drug-specific NDCs | lactulose |  |
| Drug | Drug-specific NDCs | lansoprazole |  |
| Drug | Drug-specific NDCs | levofloxacin |  |
| Drug | Drug-specific NDCs | lidocaine |  |
| Drug | Drug-specific NDCs | lisinopril |  |
| Drug | Drug-specific NDCs | loratadine |  |
| Drug | Drug-specific NDCs | meclizine hydrochloride |  |
| Drug | Drug-specific NDCs | metformin hydrochloride | Y |
| Drug | Drug-specific NDCs | methocarbamol |  |
| Drug | Drug-specific NDCs | metoclopramide hydrochloride |  |
| Drug | Drug-specific NDCs | metoprolol succinate |  |
| Drug | Drug-specific NDCs | metoprolol tartrate |  |
| Drug | Drug-specific NDCs | mometasone furoate monohydrate |  |
| Drug | Drug-specific NDCs | moxifloxacin hydrochloride |  |
| Drug | Drug-specific NDCs | niacin |  |
| Drug | Drug-specific NDCs | nifedipine |  |
| Drug | Drug-specific NDCs | nitroglycerin |  |
| Drug | Drug-specific NDCs | olopatadine hydrochloride |  |
| Drug | Drug-specific NDCs | oxycodone hydrochloride | Y |
| Drug | Drug-specific NDCs | pioglitazone hydrochloride | Y |
| Drug | Drug-specific NDCs | potassium chloride |  |
| Drug | Drug-specific NDCs | prednisone | Y |
| Drug | Drug-specific NDCs | promethazine hydrochloride | Y |
| Drug | Drug-specific NDCs | pseudoephedrine hydrochloride |  |
| Drug | Drug-specific NDCs | ramipril |  |
| Drug | Drug-specific NDCs | repaglinide |  |
| Drug | Drug-specific NDCs | rosiglitazone maleate | Y |
| Drug | Drug-specific NDCs | rosuvastatin calcium |  |
| Drug | Drug-specific NDCs | silver sulfadiazine |  |
| Drug | Drug-specific NDCs | simvastatin | Y |
| Drug | Drug-specific NDCs | sitagliptin (as phosphate) | Y |
| Drug | Drug-specific NDCs | sodium chloride |  |
| Drug | Drug-specific NDCs | spironolactone |  |
| Drug | Drug-specific NDCs | tamsulosin hydrochloride |  |
| Drug | Drug-specific NDCs | tramadol hydrochloride | Y |
| Drug | Drug-specific NDCs | warfarin sodium |  |
| Inpatient ICD-9 Dx | 038 | septicemia |  |
| Inpatient ICD-9 Dx | 041 | bact inf in oth dis/nos |  |
| Inpatient ICD-9 Dx | 244 | acquired hypothyroidism |  |
| Inpatient ICD-9 Dx | 250 | diabetes mellitus |  |
| Inpatient ICD-9 Dx | 263 | prot-cal malnutr nec/nos |  |
| Inpatient ICD-9 Dx | 272 | dis of lipoid metabolism |  |
| Inpatient ICD-9 Dx | 275 | dis mineral metabolism |  |
| Inpatient ICD-9 Dx | 276 | fluid/electrolyte dis |  |
| Inpatient ICD-9 Dx | 278 | obesity/hyperaliment |  |
| Inpatient ICD-9 Dx | 280 | iron deficiency anemias |  |
| Inpatient ICD-9 Dx | 285 | anemia nec/nos |  |
| Inpatient ICD-9 Dx | 287 | purpura & oth hemor cond |  |
| Inpatient ICD-9 Dx | 294 | other organic psych cond |  |
| Inpatient ICD-9 Dx | 305 | nondependent drug abuse |  |
| Inpatient ICD-9 Dx | 345 | epilepsy |  |
| Inpatient ICD-9 Dx | 348 | other brain conditions |  |
| Inpatient ICD-9 Dx | 357 | inflam/toxic neuropathy |  |
| Inpatient ICD-9 Dx | 401 | essential hypertension |  |
| Inpatient ICD-9 Dx | 403 | hypertensive renal dis |  |
| Inpatient ICD-9 Dx | 404 | hyperten heart/renal dis |  |
| Inpatient ICD-9 Dx | 410 | acute myocardial infarct |  |
| Inpatient ICD-9 Dx | 412 | old myocardial infarct |  |
| Inpatient ICD-9 Dx | 414 | oth chr ischemic hrt dis |  |
| Inpatient ICD-9 Dx | 416 | chr pulmonary heart dis |  |
| Inpatient ICD-9 Dx | 424 | oth endocardial disease |  |
| Inpatient ICD-9 Dx | 425 | cardiomyopathy |  |
| Inpatient ICD-9 Dx | 426 | conduction disorders |  |
| Inpatient ICD-9 Dx | 427 | cardiac dysrhythmias |  |
| Inpatient ICD-9 Dx | 428 | heart failure |  |
| Inpatient ICD-9 Dx | 438 | late eff cerebrovasc dis |  |
| Inpatient ICD-9 Dx | 440 | atherosclerosis |  |
| Inpatient ICD-9 Dx | 443 | oth periph vascular dis |  |
| Inpatient ICD-9 Dx | 458 | hypotension |  |
| Inpatient ICD-9 Dx | 486 | pneumonia |  |
| Inpatient ICD-9 Dx | 491 | chronic bronchitis |  |
| Inpatient ICD-9 Dx | 493 | asthma |  |
| Inpatient ICD-9 Dx | 496 | chr airway obstruct nec |  |
| Inpatient ICD-9 Dx | 507 | solid/liq pneumonitis |  |
| Inpatient ICD-9 Dx | 511 | pleurisy |  |
| Inpatient ICD-9 Dx | 518 | other lung diseases |  |
| Inpatient ICD-9 Dx | 530 | diseases of esophagus |  |
| Inpatient ICD-9 Dx | 578 | gastrointestinal hemorr |  |
| Inpatient ICD-9 Dx | 583 | nephritis nos |  |
| Inpatient ICD-9 Dx | 584 | acute renal failure |  |
| Inpatient ICD-9 Dx | 585 | chronic renal failure |  |
| Inpatient ICD-9 Dx | 593 | oth renal & ureteral dis |  |
| Inpatient ICD-9 Dx | 599 | oth urinary tract disor |  |
| Inpatient ICD-9 Dx | 682 | other cellulitis/abscess |  |
| Inpatient ICD-9 Dx | 707 | chronic ulcer of skin |  |
| Inpatient ICD-9 Dx | 715 | osteoarthrosis et al |  |
| Inpatient ICD-9 Dx | 729 | other soft tissue dis |  |
| Inpatient ICD-9 Dx | 780 | general symptoms |  |
| Inpatient ICD-9 Dx | 785 | cardiovascular sys symp |  |
| Inpatient ICD-9 Dx | 786 | resp sys/oth chest symp |  |
| Inpatient ICD-9 Dx | 789 | oth abdomen/pelvis symp |  |
| Inpatient ICD-9 Dx | 790 | abnormal blood findings |  |
| Inpatient ICD-9 Dx | 995 | certain adverse eff nec |  |
| Inpatient ICD-9 Dx | 996 | replace & graft complic |  |
| Inpatient ICD-9 Dx | V10 | hx of malignant neoplasm |  |
| Inpatient ICD-9 Dx | V12 | hx of disease nec |  |
| Inpatient ICD-9 Dx | V15 | oth hx of health hazards |  |
| Inpatient ICD-9 Dx | V45 | oth postsurgical states |  |
| Inpatient ICD-9 Dx | V46 | other machine dependence |  |
| Inpatient ICD-9 Dx | V49 | limb problem/problem nec |  |
| Inpatient ICD-9 Dx | V58 | encountr proc/aftrcr nec |  |
| Inpatient ICD-9 Px | 31 | larynx trachea ops nec |  |
| Inpatient ICD-9 Px | 37 | other heart/pericard ops |  |
| Inpatient ICD-9 Px | 38 | vessel inc/excis/occlus |  |
| Inpatient ICD-9 Px | 39 | other ops on vessels |  |
| Inpatient ICD-9 Px | 45 | intest incis/excis/anast |  |
| Inpatient ICD-9 Px | 86 | skin & subq operations |  |
| Inpatient ICD-9 Px | 88 | other dx radiology |  |
| Inpatient ICD-9 Px | 93 | pt |  |
| Inpatient ICD-9 Px | 96 | non-op intubat & irrigat |  |
| Inpatient ICD-9 Px | 99 | other nonoperative proc |  |
| Outpatient ICD-9 Dx | 038 | septicemia |  |
| Outpatient ICD-9 Dx | 041 | bact inf in oth dis/nos |  |
| Outpatient ICD-9 Dx | 110 | dermatophytosis |  |
| Outpatient ICD-9 Dx | 112 | candidiasis |  |
| Outpatient ICD-9 Dx | 239 | unspecified neoplasm |  |
| Outpatient ICD-9 Dx | 244 | acquired hypothyroidism | Y |
| Outpatient ICD-9 Dx | 250 | diabetes mellitus | Y |
| Outpatient ICD-9 Dx | 272 | dis of lipoid metabolism | Y |
| Outpatient ICD-9 Dx | 274 | gout |  |
| Outpatient ICD-9 Dx | 275 | dis mineral metabolism |  |
| Outpatient ICD-9 Dx | 276 | fluid/electrolyte dis |  |
| Outpatient ICD-9 Dx | 280 | iron deficiency anemias |  |
| Outpatient ICD-9 Dx | 285 | anemia nec/nos |  |
| Outpatient ICD-9 Dx | 288 | wbc disorders |  |
| Outpatient ICD-9 Dx | 290 | senile/presenile psychos |  |
| Outpatient ICD-9 Dx | 294 | other organic psych cond |  |
| Outpatient ICD-9 Dx | 296 | affective psychoses |  |
| Outpatient ICD-9 Dx | 298 | oth nonorganic psychoses |  |
| Outpatient ICD-9 Dx | 305 | nondependent drug abuse | Y |
| Outpatient ICD-9 Dx | 327 | organic sleep disorders |  |
| Outpatient ICD-9 Dx | 331 | cerebral degeneration |  |
| Outpatient ICD-9 Dx | 345 | epilepsy |  |
| Outpatient ICD-9 Dx | 348 | other brain conditions |  |
| Outpatient ICD-9 Dx | 354 | mononeuritis upper limb |  |
| Outpatient ICD-9 Dx | 356 | hered periph neuropathy |  |
| Outpatient ICD-9 Dx | 357 | inflam/toxic neuropathy |  |
| Outpatient ICD-9 Dx | 362 | retinal disorders nec | Y |
| Outpatient ICD-9 Dx | 365 | glaucoma |  |
| Outpatient ICD-9 Dx | 366 | cataract | Y |
| Outpatient ICD-9 Dx | 375 | lacrimal system disorder |  |
| Outpatient ICD-9 Dx | 380 | disorder of external ear |  |
| Outpatient ICD-9 Dx | 389 | hearing loss |  |
| Outpatient ICD-9 Dx | 401 | essential hypertension | Y |
| Outpatient ICD-9 Dx | 402 | hypertensive heart dis |  |
| Outpatient ICD-9 Dx | 403 | hypertensive renal dis |  |
| Outpatient ICD-9 Dx | 410 | acute myocardial infarct |  |
| Outpatient ICD-9 Dx | 411 | oth ac ischemic hrt dis |  |
| Outpatient ICD-9 Dx | 412 | old myocardial infarct |  |
| Outpatient ICD-9 Dx | 413 | angina pectoris |  |
| Outpatient ICD-9 Dx | 414 | oth chr ischemic hrt dis |  |
| Outpatient ICD-9 Dx | 424 | oth endocardial disease |  |
| Outpatient ICD-9 Dx | 425 | cardiomyopathy |  |
| Outpatient ICD-9 Dx | 426 | conduction disorders |  |
| Outpatient ICD-9 Dx | 427 | cardiac dysrhythmias |  |
| Outpatient ICD-9 Dx | 428 | heart failure |  |
| Outpatient ICD-9 Dx | 429 | ill-defined heart dis |  |
| Outpatient ICD-9 Dx | 433 | precerebral occlusion |  |
| Outpatient ICD-9 Dx | 434 | cerebral artery occlus |  |
| Outpatient ICD-9 Dx | 436 | cva |  |
| Outpatient ICD-9 Dx | 437 | oth cerebrovasc disease |  |
| Outpatient ICD-9 Dx | 438 | late eff cerebrovasc dis |  |
| Outpatient ICD-9 Dx | 440 | atherosclerosis |  |
| Outpatient ICD-9 Dx | 443 | oth periph vascular dis |  |
| Outpatient ICD-9 Dx | 453 | oth venous thrombosis |  |
| Outpatient ICD-9 Dx | 458 | hypotension |  |
| Outpatient ICD-9 Dx | 461 | acute sinusitis | Y |
| Outpatient ICD-9 Dx | 466 | ac bronchitis/bronchiol | Y |
| Outpatient ICD-9 Dx | 477 | allergic rhinitis |  |
| Outpatient ICD-9 Dx | 486 | pneumonia |  |
| Outpatient ICD-9 Dx | 490 | bronchitis nos |  |
| Outpatient ICD-9 Dx | 491 | chronic bronchitis |  |
| Outpatient ICD-9 Dx | 493 | asthma |  |
| Outpatient ICD-9 Dx | 496 | chr airway obstruct nec |  |
| Outpatient ICD-9 Dx | 511 | pleurisy |  |
| Outpatient ICD-9 Dx | 514 | pulm congest/hypostasis |  |
| Outpatient ICD-9 Dx | 518 | other lung diseases |  |
| Outpatient ICD-9 Dx | 530 | diseases of esophagus | Y |
| Outpatient ICD-9 Dx | 578 | gastrointestinal hemorr |  |
| Outpatient ICD-9 Dx | 584 | acute renal failure |  |
| Outpatient ICD-9 Dx | 585 | chronic renal failure |  |
| Outpatient ICD-9 Dx | 586 | renal failure nos |  |
| Outpatient ICD-9 Dx | 588 | impaired renal function |  |
| Outpatient ICD-9 Dx | 593 | oth renal & ureteral dis |  |
| Outpatient ICD-9 Dx | 599 | oth urinary tract disor |  |
| Outpatient ICD-9 Dx | 616 | oth female gen inflam |  |
| Outpatient ICD-9 Dx | 625 | female genital symptoms |  |
| Outpatient ICD-9 Dx | 627 | menopausal disorders |  |
| Outpatient ICD-9 Dx | 681 | cellulitis |  |
| Outpatient ICD-9 Dx | 682 | other cellulitis/abscess |  |
| Outpatient ICD-9 Dx | 703 | diseases of nail |  |
| Outpatient ICD-9 Dx | 707 | chronic ulcer of skin |  |
| Outpatient ICD-9 Dx | 715 | osteoarthrosis et al | Y |
| Outpatient ICD-9 Dx | 719 | joint disorder nec & nos |  |
| Outpatient ICD-9 Dx | 724 | back disorder nec & nos | Y |
| Outpatient ICD-9 Dx | 726 | periph enthesopathies |  |
| Outpatient ICD-9 Dx | 727 | oth dis synov/tend/bursa |  |
| Outpatient ICD-9 Dx | 728 | dis of muscle/lig/fascia |  |
| Outpatient ICD-9 Dx | 729 | other soft tissue dis |  |
| Outpatient ICD-9 Dx | 733 | oth bone & cartilage dis | Y |
| Outpatient ICD-9 Dx | 780 | general symptoms |  |
| Outpatient ICD-9 Dx | 781 | nerv/musculskel sys symp |  |
| Outpatient ICD-9 Dx | 782 | skin/oth integument symp | Y |
| Outpatient ICD-9 Dx | 783 | nutrit/metab/devel symp |  |
| Outpatient ICD-9 Dx | 784 | symptoms invol head/neck |  |
| Outpatient ICD-9 Dx | 785 | cardiovascular sys symp |  |
| Outpatient ICD-9 Dx | 786 | resp sys/oth chest symp |  |
| Outpatient ICD-9 Dx | 787 | gi system symptoms |  |
| Outpatient ICD-9 Dx | 788 | urinary system symptoms |  |
| Outpatient ICD-9 Dx | 789 | oth abdomen/pelvis symp |  |
| Outpatient ICD-9 Dx | 790 | abnormal blood findings | Y |
| Outpatient ICD-9 Dx | 793 | abn find-body struct nos |  |
| Outpatient ICD-9 Dx | 794 | abnormal function study |  |
| Outpatient ICD-9 Dx | 796 | other abnormal findings |  |
| Outpatient ICD-9 Dx | 799 | oth ill-def morbid/mortl |  |
| Outpatient ICD-9 Dx | 840 | sprain shoulder & arm |  |
| Outpatient ICD-9 Dx | 995 | certain adverse eff nec |  |
| Outpatient ICD-9 Dx | 996 | replace & graft complic |  |
| Outpatient ICD-9 Dx | 998 | oth surgical compl nec |  |
| Outpatient ICD-9 Dx | V03 | vaccin for bacterial dis | Y |
| Outpatient ICD-9 Dx | V04 | vaccin for viral disease | Y |
| Outpatient ICD-9 Dx | V12 | hx of disease nec |  |
| Outpatient ICD-9 Dx | V15 | oth hx of health hazards |  |
| Outpatient ICD-9 Dx | V43 | organ replacement nec |  |
| Outpatient ICD-9 Dx | V45 | oth postsurgical states |  |
| Outpatient ICD-9 Dx | V57 | rehabilitation procedure |  |
| Outpatient ICD-9 Dx | V58 | encountr proc/aftrcr nec |  |
| Outpatient ICD-9 Dx | V71 | observation-suspect cond |  |
| Outpatient ICD-9 Dx | V76 | screening-malig neoplasm | Y |
| Outpatient ICD-9 Px | 39 | other ops on vessels |  |
| Outpatient CPT-4 Px | 11721 | debride nail, 6 or more |  |
| Outpatient CPT-4 Px | 20610 | drain/inject, joint/bursa |  |
| Outpatient CPT-4 Px | 36415 | routine venipuncture | Y |
| Outpatient CPT-4 Px | 70450 | ct head/brain w/o dye |  |
| Outpatient CPT-4 Px | 71010 | chest x-ray |  |
| Outpatient CPT-4 Px | 71020 | chest x-ray |  |
| Outpatient CPT-4 Px | 71260 | ct thorax w/dye |  |
| Outpatient CPT-4 Px | 72192 | ct pelvis w/o dye |  |
| Outpatient CPT-4 Px | 73630 | x-ray foot |  |
| Outpatient CPT-4 Px | 74000 | x-ray abdomen |  |
| Outpatient CPT-4 Px | 74150 | ct abdomen w/o dye |  |
| Outpatient CPT-4 Px | 74160 | ct abdomen w/dye |  |
| Outpatient CPT-4 Px | 76700 | us exam abdom, complete |  |
| Outpatient CPT-4 Px | 76770 | us exam abdo back wall, comp |  |
| Outpatient CPT-4 Px | 77052 | comp screen mammogram add-on |  |
| Outpatient CPT-4 Px | 78465 | heart image (3d), multiple |  |
| Outpatient CPT-4 Px | 78478 | heart wall motion add-on |  |
| Outpatient CPT-4 Px | 78480 | heart function add-on |  |
| Outpatient CPT-4 Px | 80048 | metabolic panel total ca |  |
| Outpatient CPT-4 Px | 80051 | electrolyte panel |  |
| Outpatient CPT-4 Px | 80053 | comprehen metabolic panel |  |
| Outpatient CPT-4 Px | 80061 | lipid panel | Y |
| Outpatient CPT-4 Px | 80076 | hepatic function panel |  |
| Outpatient CPT-4 Px | 81001 | urinalysis, auto w/scope |  |
| Outpatient CPT-4 Px | 82040 | assay serum albumin |  |
| Outpatient CPT-4 Px | 82043 | microalbumin, quantitative | Y |
| Outpatient CPT-4 Px | 82150 | assay amylase |  |
| Outpatient CPT-4 Px | 82248 | bilirubin, direct |  |
| Outpatient CPT-4 Px | 82310 | assay calcium |  |
| Outpatient CPT-4 Px | 82550 | assay ck (cpk) |  |
| Outpatient CPT-4 Px | 82553 | creatine, mb fraction |  |
| Outpatient CPT-4 Px | 82565 | assay creatinine | Y |
| Outpatient CPT-4 Px | 82607 | vitamin b-12 |  |
| Outpatient CPT-4 Px | 82728 | assay ferritin |  |
| Outpatient CPT-4 Px | 82746 | blood folic acid serum |  |
| Outpatient CPT-4 Px | 82947 | assay glucose, blood quant |  |
| Outpatient CPT-4 Px | 82962 | glucose blood test |  |
| Outpatient CPT-4 Px | 82977 | assay ggt | Y |
| Outpatient CPT-4 Px | 83036 | glycosylated hemoglobin test |  |
| Outpatient CPT-4 Px | 83540 | assay iron |  |
| Outpatient CPT-4 Px | 83550 | iron binding test |  |
| Outpatient CPT-4 Px | 83615 | lactate (ld) (ldh) enzyme |  |
| Outpatient CPT-4 Px | 83718 | assay lipoprotein |  |
| Outpatient CPT-4 Px | 83735 | assay magnesium |  |
| Outpatient CPT-4 Px | 83880 | natriuretic peptide |  |
| Outpatient CPT-4 Px | 83970 | assay parathormone |  |
| Outpatient CPT-4 Px | 84100 | assay phosphorus |  |
| Outpatient CPT-4 Px | 84132 | assay serum potassium |  |
| Outpatient CPT-4 Px | 84153 | assay psa, total | Y |
| Outpatient CPT-4 Px | 84436 | assay total thyroxine | Y |
| Outpatient CPT-4 Px | 84439 | assay free thyroxine | Y |
| Outpatient CPT-4 Px | 84443 | assay thyroid stim hormone | Y |
| Outpatient CPT-4 Px | 84450 | transferase (ast) (sgot) |  |
| Outpatient CPT-4 Px | 84460 | alanine amino (alt) (sgpt) |  |
| Outpatient CPT-4 Px | 84484 | assay troponin, quant |  |
| Outpatient CPT-4 Px | 84520 | assay urea nitrogen | Y |
| Outpatient CPT-4 Px | 84550 | assay blood/uric acid |  |
| Outpatient CPT-4 Px | 85024 | partial cbc w/ auto diff wbc | Y |
| Outpatient CPT-4 Px | 85025 | complete cbc w/auto diff wbc |  |
| Outpatient CPT-4 Px | 85027 | complete cbc, automated |  |
| Outpatient CPT-4 Px | 85610 | prothrombin time |  |
| Outpatient CPT-4 Px | 85651 | rbc sed rate, nonautomated | Y |
| Outpatient CPT-4 Px | 85730 | thromboplastin time, partial |  |
| Outpatient CPT-4 Px | 86677 | helicobacter pylori |  |
| Outpatient CPT-4 Px | 86706 | hep b surface antibody |  |
| Outpatient CPT-4 Px | 87040 | blood culture for bacteria |  |
| Outpatient CPT-4 Px | 87070 | culture, bacteria, other |  |
| Outpatient CPT-4 Px | 87077 | culture aerobic identify |  |
| Outpatient CPT-4 Px | 87086 | urine culture/colony count | Y |
| Outpatient CPT-4 Px | 87088 | urine bacteria culture |  |
| Outpatient CPT-4 Px | 87186 | microbe susceptible, mic |  |
| Outpatient CPT-4 Px | 87340 | hepatitis b surface ag, eia |  |
| Outpatient CPT-4 Px | 88142 | cytopath, c/v, thin layer |  |
| Outpatient CPT-4 Px | 88305 | tissue exam by pathologist | Y |
| Outpatient CPT-4 Px | 90471 | immunization admin |  |
| Outpatient CPT-4 Px | 90801 | psych dx interview | Y |
| Outpatient CPT-4 Px | 92012 | eye exam established pat |  |
| Outpatient CPT-4 Px | 92014 | eye exam & treat |  |
| Outpatient CPT-4 Px | 92135 | ophth dx imaging post seg |  |
| Outpatient CPT-4 Px | 92226 | special eye exam subsequent |  |
| Outpatient CPT-4 Px | 93000 | ecg complete | Y |
| Outpatient CPT-4 Px | 93005 | ecg tracing |  |
| Outpatient CPT-4 Px | 93010 | ecg report |  |
| Outpatient CPT-4 Px | 93015 | cardiovascular stress test |  |
| Outpatient CPT-4 Px | 93016 | cardiovascular stress test |  |
| Outpatient CPT-4 Px | 93018 | cardiovascular stress test | Y |
| Outpatient CPT-4 Px | 93042 | rhythm ecg, report |  |
| Outpatient CPT-4 Px | 93306 | echocardiography, transthoracic, real-time with image documentation |  |
| Outpatient CPT-4 Px | 93307 | echo exam heart |  |
| Outpatient CPT-4 Px | 93320 | doppler echo exam heart |  |
| Outpatient CPT-4 Px | 93325 | doppler color flow add-on |  |
| Outpatient CPT-4 Px | 93545 | inject for coronary x-rays |  |
| Outpatient CPT-4 Px | 93556 | imaging, cardiac cath |  |
| Outpatient CPT-4 Px | 93880 | extracranial study |  |
| Outpatient CPT-4 Px | 93970 | extremity study |  |
| Outpatient CPT-4 Px | 94010 | breathing capacity test |  |
| Outpatient CPT-4 Px | 94060 | evaluate wheezing |  |
| Outpatient CPT-4 Px | 94640 | airway inhalation treat |  |
| Outpatient CPT-4 Px | 97001 | pt evaluation | Y |
| Outpatient CPT-4 Px | 97035 | ultrasound therapy |  |
| Outpatient CPT-4 Px | 99212 | office/outpatient visit, est | Y |
| Outpatient CPT-4 Px | 99213 | office/outpatient visit, est | Y |
| Outpatient CPT-4 Px | 99214 | office/outpatient visit, est |  |
| Outpatient CPT-4 Px | 99215 | office/outpatient visit, est |  |
| Outpatient CPT-4 Px | 99222 | initial hospital care |  |
| Outpatient CPT-4 Px | 99223 | initial hospital care |  |
| Outpatient CPT-4 Px | 99231 | subsequent hospital care |  |
| Outpatient CPT-4 Px | 99232 | subsequent hospital care |  |
| Outpatient CPT-4 Px | 99233 | subsequent hospital care |  |
| Outpatient CPT-4 Px | 99238 | hospital discharge day |  |
| Outpatient CPT-4 Px | 99239 | hospital discharge day |  |
| Outpatient CPT-4 Px | 99244 | office consultation | Y |
| Outpatient CPT-4 Px | 99245 | office consultation |  |
| Outpatient CPT-4 Px | 99253 | inpatient consultation |  |
| Outpatient CPT-4 Px | 99254 | inpatient consultation |  |
| Outpatient CPT-4 Px | 99255 | inpatient consultation |  |
| Outpatient CPT-4 Px | 99283 | emergency dept visit | Y |
| Outpatient CPT-4 Px | 99284 | emergency dept visit |  |
| Outpatient CPT-4 Px | 99285 | emergency dept visit |  |
| Outpatient CPT-4 Px | 99291 | critical care, first hour |  |
| Outpatient CPT-4 Px | A0425 | ground mileage, per statute mile |  |
| Outpatient CPT-4 Px | A0427 | ambulance service, advanced life support, emergency transport |  |
| Outpatient CPT-4 Px | A0428 | ambulance service, basic life support, non-emergency transport |  |
| Outpatient CPT-4 Px | A0429 | ambulance service, basic life support, emergency transport |  |
| Outpatient CPT-4 Px | D0110 | initial oral examination | Y |
| Outpatient CPT-4 Px | D0120 | periodic oral examination | Y |
| Outpatient CPT-4 Px | D0230 | intraoral-periapical | Y |
| Outpatient CPT-4 Px | D0272 | bitewings-two films |  |
| Outpatient CPT-4 Px | D0274 | bitewings-four films |  |
| Outpatient CPT-4 Px | D1110 | dental prophylaxis-adults | Y |
| Outpatient CPT-4 Px | G0001 | routine venipuncture for collection of specimen |  |
| Outpatient CPT-4 Px | G0008 | admin influenza virus vac |  |
| Outpatient CPT-4 Px | G0202 | screening mammography, bilateral |  |
| Outpatient CPT-4 Px | X0030 | ambulance service, basic life support base rate, emergency transport, one way |  |
| Outpatient CPT-4 Px | X0034 | ambulance service, basic life support, per mile, transport, one way |  |
| Outpatient CPT-4 Px | X7700 | administered intravenous solution | Y |
| Outpatient CPT-4 Px | Z2932 | dispensing fees, bifocal lens | Y |
| Outpatient CPT-4 Px | Z3136 | pneumococcal antibody panel | Y |
| Outpatient CPT-4 Px | Z7502 | use of emergency room | Y |
| Outpatient CPT-4 Px | Z7610 | misc drugs and med supplies, admin stat | Y |
| Outpatient CPT-4 Px | Z9525 | personal care services program |  |
| Other Setting ICD-9 Dx | 250 | diabetes mellitus |  |
| Other Setting ICD-9 Dx | 272 | dis of lipoid metabolism |  |
| Other Setting ICD-9 Dx | 285 | anemia nec/nos |  |
| Other Setting ICD-9 Dx | 401 | essential hypertension |  |
| Other Setting ICD-9 Dx | 414 | oth chr ischemic hrt dis |  |
| Other Setting ICD-9 Dx | 428 | heart failure |  |
| Other Setting ICD-9 Dx | 438 | late eff cerebrovasc dis |  |
| Other Setting ICD-9 Dx | 496 | chr airway obstruct nec |  |
| Other Setting ICD-9 Dx | 518 | other lung diseases |  |
| Other Setting ICD-9 Dx | 585 | chronic renal failure |  |
| Other Setting ICD-9 Dx | 682 | other cellulitis/abscess |  |
| Other Setting ICD-9 Dx | 728 | dis of muscle/lig/fascia |  |
| Other Setting ICD-9 Dx | 746 | other congen heart anom |  |
| Other Setting ICD-9 Dx | 780 | general symptoms |  |
| Other Setting ICD-9 Dx | 787 | gi system symptoms |  |
| CPT-4 = Current Procedural Terminology; Dx = diagnosis; ICD-9 = International Classification of Diseases, 9th Revision; NDC = National Drug Code; Px = procedure  * In a pre-specified secondary analysis, covariates with z-bias=5 were excluded from the propensity score as they were strongly related to exposure, but not outcome | | | |

**Supplementary Table 5. Empirically identified covariates included in propensity score (Optum)**

| **Data Dimension** | **Code** | **Code description** | **Z-bias = 5*** |
| --- | --- | --- | --- |
| Drug | Drug-specific NDCs | albuterol |  |
| Drug | Drug-specific NDCs | amlodipine besylate | Y |
| Drug | Drug-specific NDCs | amoxicillin (as trihydrate) | Y |
| Drug | Drug-specific NDCs | benzonatate |  |
| Drug | Drug-specific NDCs | bupropion hydrochloride |  |
| Drug | Drug-specific NDCs | carvedilol |  |
| Drug | Drug-specific NDCs | clavulanate potassium |  |
| Drug | Drug-specific NDCs | clopidogrel bisulfate |  |
| Drug | Drug-specific NDCs | diclofenac sodium |  |
| Drug | Drug-specific NDCs | digoxin |  |
| Drug | Drug-specific NDCs | ergocalciferol |  |
| Drug | Drug-specific NDCs | escitalopram oxalate |  |
| Drug | Drug-specific NDCs | estradiol |  |
| Drug | Drug-specific NDCs | exenatide |  |
| Drug | Drug-specific NDCs | fenofibrate, micronized |  |
| Drug | Drug-specific NDCs | fluconazole |  |
| Drug | Drug-specific NDCs | furosemide |  |
| Drug | Drug-specific NDCs | gabapentin |  |
| Drug | Drug-specific NDCs | hydralazine hydrochloride |  |
| Drug | Drug-specific NDCs | hydrochlorothiazide |  |
| Drug | Drug-specific NDCs | hydrocortisone |  |
| Drug | Drug-specific NDCs | insulin aspart |  |
| Drug | Drug-specific NDCs | insulin detemir |  |
| Drug | Drug-specific NDCs | insulin glargine |  |
| Drug | Drug-specific NDCs | insulin lispro |  |
| Drug | Drug-specific NDCs | isosorbide mononitrate |  |
| Drug | Drug-specific NDCs | liraglutide |  |
| Drug | Drug-specific NDCs | losartan potassium | Y |
| Drug | Drug-specific NDCs | metformin hydrochloride | Y |
| Drug | Drug-specific NDCs | methylprednisolone |  |
| Drug | Drug-specific NDCs | metoprolol succinate |  |
| Drug | Drug-specific NDCs | metoprolol tartrate |  |
| Drug | Drug-specific NDCs | nitroglycerin |  |
| Drug | Drug-specific NDCs | olmesartan medoxomil |  |
| Drug | Drug-specific NDCs | omeprazole | Y |
| Drug | Drug-specific NDCs | oxycodone hydrochloride |  |
| Drug | Drug-specific NDCs | pioglitazone hydrochloride |  |
| Drug | Drug-specific NDCs | potassium chloride |  |
| Drug | Drug-specific NDCs | promethazine hydrochloride |  |
| Drug | Drug-specific NDCs | propranolol hydrochloride |  |
| Drug | Drug-specific NDCs | rosiglitazone maleate |  |
| Drug | Drug-specific NDCs | rosuvastatin calcium |  |
| Drug | Drug-specific NDCs | saxagliptin |  |
| Drug | Drug-specific NDCs | simvastatin | Y |
| Drug | Drug-specific NDCs | sitagliptin |  |
| Drug | Drug-specific NDCs | sitagliptin (as phosphate) | Y |
| Drug | Drug-specific NDCs | spironolactone |  |
| Drug | Drug-specific NDCs | sulfamethoxazole |  |
| Drug | Drug-specific NDCs | tadalafil |  |
| Drug | Drug-specific NDCs | tamsulosin hydrochloride |  |
| Drug | Drug-specific NDCs | tiotropium |  |
| Drug | Drug-specific NDCs | trimethoprim |  |
| Drug | Drug-specific NDCs | warfarin sodium |  |
| Inpatient ICD-9 Dx | 038 | septicemia |  |
| Inpatient ICD-9 Dx | 162 | mal neo trachea/lung |  |
| Inpatient ICD-9 Dx | 197 | secondry mal neo gi/resp |  |
| Inpatient ICD-9 Dx | 198 | sec malig neo oth sites |  |
| Inpatient ICD-9 Dx | 250 | diabetes mellitus |  |
| Inpatient ICD-9 Dx | 272 | dis of lipoid metabolism |  |
| Inpatient ICD-9 Dx | 275 | dis mineral metabolism |  |
| Inpatient ICD-9 Dx | 276 | fluid/electrolyte dis |  |
| Inpatient ICD-9 Dx | 280 | iron deficiency anemias |  |
| Inpatient ICD-9 Dx | 284 | aplastic anemia |  |
| Inpatient ICD-9 Dx | 285 | anemia nec/nos |  |
| Inpatient ICD-9 Dx | 287 | purpura & oth hemor cond |  |
| Inpatient ICD-9 Dx | 288 | wbc disorders |  |
| Inpatient ICD-9 Dx | 305 | nondependent drug abuse |  |
| Inpatient ICD-9 Dx | 338 | pain, not elsewhere classified |  |
| Inpatient ICD-9 Dx | 348 | other brain conditions |  |
| Inpatient ICD-9 Dx | 357 | inflam/toxic neuropathy |  |
| Inpatient ICD-9 Dx | 401 | essential hypertension |  |
| Inpatient ICD-9 Dx | 403 | hypertensive renal dis |  |
| Inpatient ICD-9 Dx | 410 | acute myocardial infarct |  |
| Inpatient ICD-9 Dx | 414 | oth chr ischemic hrt dis |  |
| Inpatient ICD-9 Dx | 416 | chr pulmonary heart dis |  |
| Inpatient ICD-9 Dx | 424 | oth endocardial disease |  |
| Inpatient ICD-9 Dx | 425 | cardiomyopathy |  |
| Inpatient ICD-9 Dx | 426 | conduction disorders |  |
| Inpatient ICD-9 Dx | 427 | cardiac dysrhythmias |  |
| Inpatient ICD-9 Dx | 428 | heart failure |  |
| Inpatient ICD-9 Dx | 429 | ill-defined heart dis |  |
| Inpatient ICD-9 Dx | 443 | oth periph vascular dis |  |
| Inpatient ICD-9 Dx | 458 | hypotension |  |
| Inpatient ICD-9 Dx | 459 | oth circulatory disease |  |
| Inpatient ICD-9 Dx | 486 | pneumonia |  |
| Inpatient ICD-9 Dx | 491 | chronic bronchitis |  |
| Inpatient ICD-9 Dx | 496 | chr airway obstruct nec |  |
| Inpatient ICD-9 Dx | 511 | pleurisy |  |
| Inpatient ICD-9 Dx | 514 | pulm congest/hypostasis |  |
| Inpatient ICD-9 Dx | 518 | other lung diseases |  |
| Inpatient ICD-9 Dx | 578 | gastrointestinal hemorr |  |
| Inpatient ICD-9 Dx | 584 | acute renal failure |  |
| Inpatient ICD-9 Dx | 585 | chronic renal failure |  |
| Inpatient ICD-9 Dx | 593 | oth renal & ureteral dis |  |
| Inpatient ICD-9 Dx | 599 | oth urinary tract disor |  |
| Inpatient ICD-9 Dx | 682 | other cellulitis/abscess |  |
| Inpatient ICD-9 Dx | 707 | chronic ulcer of skin |  |
| Inpatient ICD-9 Dx | 729 | other soft tissue dis |  |
| Inpatient ICD-9 Dx | 780 | general symptoms |  |
| Inpatient ICD-9 Dx | 782 | skin/oth integument symp |  |
| Inpatient ICD-9 Dx | 784 | symptoms invol head/neck |  |
| Inpatient ICD-9 Dx | 785 | cardiovascular sys symp |  |
| Inpatient ICD-9 Dx | 786 | resp sys/oth chest symp |  |
| Inpatient ICD-9 Dx | 787 | gi system symptoms |  |
| Inpatient ICD-9 Dx | 790 | abnormal blood findings |  |
| Inpatient ICD-9 Dx | 793 | abn find-body struct nos |  |
| Inpatient ICD-9 Dx | 794 | abnormal function study |  |
| Inpatient ICD-9 Dx | 799 | oth ill-def morbid/mortl |  |
| Inpatient ICD-9 Dx | 995 | certain adverse eff nec |  |
| Inpatient ICD-9 Dx | 996 | replace & graft complic |  |
| Inpatient ICD-9 Dx | V12 | hx of disease nec |  |
| Inpatient ICD-9 Dx | V15 | oth hx of health hazards |  |
| Inpatient ICD-9 Dx | V45 | oth postsurgical states |  |
| Inpatient ICD-9 Dx | V46 | other machine dependence |  |
| Inpatient ICD-9 Dx | V58 | encountr proc/aftrcr nec |  |
| Inpatient ICD-9 Dx | V85 | body mass index 45.0-49.9 |  |
| Inpatient ICD-10 Dx | D64 | other anemias |  |
| Inpatient ICD-10 Dx | E11 | type 2 diabetes mellitus |  |
| Inpatient ICD-10 Dx | E78 | disorders of lipoprotein metabolism and other lipidemias |  |
| Inpatient ICD-10 Dx | E83 | disorders of mineral metabolism |  |
| Inpatient ICD-10 Dx | E87 | other disorders of fluid, electrolyte and acid-base balance |  |
| Inpatient ICD-10 Dx | I10 | essential (primary) hypertension |  |
| Inpatient ICD-10 Dx | I42 | cardiomyopathy |  |
| Inpatient ICD-10 Dx | I48 | atrial fibrillation and flutter |  |
| Inpatient ICD-10 Dx | I50 | heart failure |  |
| Inpatient ICD-10 Dx | I73 | other peripheral vascular diseases |  |
| Inpatient ICD-10 Dx | J18 | pneumonia, unspecified organism |  |
| Inpatient ICD-10 Dx | J44 | other chronic obstructive pulmonary disease |  |
| Inpatient ICD-10 Dx | J81 | pulmonary edema |  |
| Inpatient ICD-10 Dx | J90 | pleural effusion, not elsewhere classified |  |
| Inpatient ICD-10 Dx | J96 | respiratory failure, not elsewhere classified |  |
| Inpatient ICD-10 Dx | J98 | other respiratory disorders |  |
| Inpatient ICD-10 Dx | L02 | cutaneous abscess, furuncle and carbuncle |  |
| Inpatient ICD-10 Dx | L03 | cellulitis and acute lymphangitis |  |
| Inpatient ICD-10 Dx | M19 | other and unspecified osteoarthritis |  |
| Inpatient ICD-10 Dx | M79 | other and unspecified soft tissue disorders, not elsewhere classified |  |
| Inpatient ICD-10 Dx | N18 | chronic kidney disease (ckd) |  |
| Inpatient ICD-10 Dx | R06 | abnormalities of breathing |  |
| Inpatient ICD-10 Dx | R60 | edema, not elsewhere classified |  |
| Inpatient ICD-10 Dx | R91 | abnormal findings on diagnostic imaging of lung |  |
| Inpatient ICD-10 Dx | Z79 | long term (current) drug therapy |  |
| Inpatient ICD-10 Dx | Z95 | presence of cardiac and vascular implants and grafts |  |
| Inpatient ICD-10 Dx | Z99 | dependence on enabling machines and devices, not elsewhere classified |  |
| Inpatient ICD-9 Px | 37 | other heart/pericard ops |  |
| Inpatient ICD-9 Px | 55 | operations on kidney |  |
| Inpatient ICD-9 Px | 88 | other dx radiology |  |
| Inpatient ICD-9 Px | 93 | pt |  |
| Inpatient ICD-9 Px | 99 | other nonoperative proc |  |
| Inpatient ICD-10 Px | 02H | insertion |  |
| Inpatient CPT-4 Px | 4048F | doc antibio given b/4 surg |  |
| Inpatient CPT-4 Px | 70450 | ct head/brain w/o dye |  |
| Inpatient CPT-4 Px | 71010 | chest x-ray |  |
| Inpatient CPT-4 Px | 71020 | chest x-ray |  |
| Inpatient CPT-4 Px | 71250 | ct thorax w/o dye |  |
| Inpatient CPT-4 Px | 78306 | bone imaging, whole body |  |
| Inpatient CPT-4 Px | 78452 | myocardial perfusion imaging, tomographic |  |
| Inpatient CPT-4 Px | 88305 | tissue exam by pathologist |  |
| Inpatient CPT-4 Px | 88342 | immunohistochemistry |  |
| Inpatient CPT-4 Px | 90935 | hemodialysis, one evaluation |  |
| Inpatient CPT-4 Px | 93010 | ecg report |  |
| Inpatient CPT-4 Px | 93306 | echocardiography, transthoracic, real-time with image documentation |  |
| Inpatient CPT-4 Px | 93307 | echo exam heart |  |
| Inpatient CPT-4 Px | 93320 | doppler echo exam heart |  |
| Inpatient CPT-4 Px | 93325 | doppler color flow add-on |  |
| Inpatient CPT-4 Px | 93458 | catheter placement in coronary artery(s) for coronary angiography |  |
| Inpatient CPT-4 Px | 93970 | extremity study |  |
| Inpatient CPT-4 Px | 93971 | extremity study |  |
| Inpatient CPT-4 Px | 99221 | initial hospital care |  |
| Inpatient CPT-4 Px | 99222 | initial hospital care |  |
| Inpatient CPT-4 Px | 99223 | initial hospital care |  |
| Inpatient CPT-4 Px | 99231 | subsequent hospital care |  |
| Inpatient CPT-4 Px | 99232 | subsequent hospital care |  |
| Inpatient CPT-4 Px | 99233 | subsequent hospital care |  |
| Inpatient CPT-4 Px | 99238 | hospital discharge day |  |
| Inpatient CPT-4 Px | 99239 | hospital discharge day |  |
| Inpatient CPT-4 Px | 99254 | inpatient consultation |  |
| Inpatient CPT-4 Px | 99255 | inpatient consultation |  |
| Inpatient CPT-4 Px | 99291 | critical care, first hour |  |
| Outpatient ICD-9 Dx | 238 | unc behav neo nec/nos |  |
| Outpatient ICD-9 Dx | 241 | nontoxic nodular goiter |  |
| Outpatient ICD-9 Dx | 244 | acquired hypothyroidism | Y |
| Outpatient ICD-9 Dx | 250 | diabetes mellitus | Y |
| Outpatient ICD-9 Dx | 268 | vitamin d deficiency | Y |
| Outpatient ICD-9 Dx | 272 | dis of lipoid metabolism | Y |
| Outpatient ICD-9 Dx | 276 | fluid/electrolyte dis |  |
| Outpatient ICD-9 Dx | 280 | iron deficiency anemias |  |
| Outpatient ICD-9 Dx | 285 | anemia nec/nos |  |
| Outpatient ICD-9 Dx | 288 | wbc disorders |  |
| Outpatient ICD-9 Dx | 300 | neurotic disorders |  |
| Outpatient ICD-9 Dx | 305 | nondependent drug abuse |  |
| Outpatient ICD-9 Dx | 327 | organic sleep disorders |  |
| Outpatient ICD-9 Dx | 338 | pain, not elsewhere classified |  |
| Outpatient ICD-9 Dx | 354 | mononeuritis upper limb |  |
| Outpatient ICD-9 Dx | 355 | mononeuritis leg |  |
| Outpatient ICD-9 Dx | 357 | inflam/toxic neuropathy |  |
| Outpatient ICD-9 Dx | 362 | retinal disorders nec |  |
| Outpatient ICD-9 Dx | 366 | cataract |  |
| Outpatient ICD-9 Dx | 381 | nonsuppur otitis media |  |
| Outpatient ICD-9 Dx | 401 | essential hypertension | Y |
| Outpatient ICD-9 Dx | 403 | hypertensive renal dis |  |
| Outpatient ICD-9 Dx | 412 | old myocardial infarct |  |
| Outpatient ICD-9 Dx | 414 | oth chr ischemic hrt dis |  |
| Outpatient ICD-9 Dx | 424 | oth endocardial disease |  |
| Outpatient ICD-9 Dx | 425 | cardiomyopathy |  |
| Outpatient ICD-9 Dx | 427 | cardiac dysrhythmias |  |
| Outpatient ICD-9 Dx | 428 | heart failure |  |
| Outpatient ICD-9 Dx | 429 | ill-defined heart dis |  |
| Outpatient ICD-9 Dx | 433 | precerebral occlusion |  |
| Outpatient ICD-9 Dx | 440 | atherosclerosis |  |
| Outpatient ICD-9 Dx | 443 | oth periph vascular dis |  |
| Outpatient ICD-9 Dx | 459 | oth circulatory disease |  |
| Outpatient ICD-9 Dx | 461 | acute sinusitis |  |
| Outpatient ICD-9 Dx | 466 | ac bronchitis/bronchiol |  |
| Outpatient ICD-9 Dx | 473 | chronic sinusitis |  |
| Outpatient ICD-9 Dx | 491 | chronic bronchitis |  |
| Outpatient ICD-9 Dx | 496 | chr airway obstruct nec |  |
| Outpatient ICD-9 Dx | 511 | pleurisy |  |
| Outpatient ICD-9 Dx | 518 | other lung diseases |  |
| Outpatient ICD-9 Dx | 530 | diseases of esophagus | Y |
| Outpatient ICD-9 Dx | 584 | acute renal failure |  |
| Outpatient ICD-9 Dx | 585 | chronic renal failure |  |
| Outpatient ICD-9 Dx | 593 | oth renal & ureteral dis |  |
| Outpatient ICD-9 Dx | 625 | female genital symptoms |  |
| Outpatient ICD-9 Dx | 627 | menopausal disorders |  |
| Outpatient ICD-9 Dx | 702 | other dermatoses |  |
| Outpatient ICD-9 Dx | 709 | other skin disorders |  |
| Outpatient ICD-9 Dx | 715 | osteoarthrosis et al |  |
| Outpatient ICD-9 Dx | 719 | joint disorder nec & nos |  |
| Outpatient ICD-9 Dx | 721 | spondylosis et al |  |
| Outpatient ICD-9 Dx | 722 | intervertebral disc dis |  |
| Outpatient ICD-9 Dx | 723 | other cervical spine dis |  |
| Outpatient ICD-9 Dx | 724 | back disorder nec & nos |  |
| Outpatient ICD-9 Dx | 726 | periph enthesopathies |  |
| Outpatient ICD-9 Dx | 728 | dis of muscle/lig/fascia |  |
| Outpatient ICD-9 Dx | 729 | other soft tissue dis | Y |
| Outpatient ICD-9 Dx | 733 | oth bone & cartilage dis |  |
| Outpatient ICD-9 Dx | 780 | general symptoms | Y |
| Outpatient ICD-9 Dx | 781 | nerv/musculskel sys symp |  |
| Outpatient ICD-9 Dx | 782 | skin/oth integument symp | Y |
| Outpatient ICD-9 Dx | 785 | cardiovascular sys symp |  |
| Outpatient ICD-9 Dx | 786 | resp sys/oth chest symp |  |
| Outpatient ICD-9 Dx | 790 | abnormal blood findings |  |
| Outpatient ICD-9 Dx | 793 | abn find-body struct nos |  |
| Outpatient ICD-9 Dx | 794 | abnormal function study |  |
| Outpatient ICD-9 Dx | 799 | oth ill-def morbid/mortl |  |
| Outpatient ICD-9 Dx | 847 | sprain of back nec/nos |  |
| Outpatient ICD-9 Dx | V04 | vaccin for viral disease |  |
| Outpatient ICD-9 Dx | V10 | hx of malignant neoplasm |  |
| Outpatient ICD-9 Dx | V12 | hx of disease nec |  |
| Outpatient ICD-9 Dx | V15 | oth hx of health hazards |  |
| Outpatient ICD-9 Dx | V43 | organ replacement nec |  |
| Outpatient ICD-9 Dx | V45 | oth postsurgical states |  |
| Outpatient ICD-9 Dx | V57 | rehabilitation procedure |  |
| Outpatient ICD-9 Dx | V58 | encountr proc/aftrcr nec |  |
| Outpatient ICD-9 Dx | V76 | screening-malig neoplasm | Y |
| Outpatient ICD-10 Dx | B35 | dermatophytosis |  |
| Outpatient ICD-10 Dx | D64 | other anemias |  |
| Outpatient ICD-10 Dx | E10 | type 1 diabetes mellitus |  |
| Outpatient ICD-10 Dx | E11 | type 2 diabetes mellitus | Y |
| Outpatient ICD-10 Dx | E55 | vitamin d deficiency |  |
| Outpatient ICD-10 Dx | E78 | disorders of lipoprotein metabolism and other lipidemias | Y |
| Outpatient ICD-10 Dx | E87 | other disorders of fluid, electrolyte and acid-base balance |  |
| Outpatient ICD-10 Dx | G47 | sleep disorders |  |
| Outpatient ICD-10 Dx | I10 | essential (primary) hypertension |  |
| Outpatient ICD-10 Dx | I25 | chronic ischemic heart disease |  |
| Outpatient ICD-10 Dx | I42 | cardiomyopathy |  |
| Outpatient ICD-10 Dx | I48 | atrial fibrillation and flutter |  |
| Outpatient ICD-10 Dx | I50 | heart failure |  |
| Outpatient ICD-10 Dx | I73 | other peripheral vascular diseases |  |
| Outpatient ICD-10 Dx | I87 | other disorders of veins |  |
| Outpatient ICD-10 Dx | J44 | other chronic obstructive pulmonary disease |  |
| Outpatient ICD-10 Dx | L97 | non-pressure chronic ulcer of lower limb, not elsewhere classified |  |
| Outpatient ICD-10 Dx | M54 | dorsalgia |  |
| Outpatient ICD-10 Dx | M62 | other disorders of muscle |  |
| Outpatient ICD-10 Dx | M79 | other and unspecified soft tissue disorders, not elsewhere classified |  |
| Outpatient ICD-10 Dx | N28 | other disorders of kidney and ureter, not elsewhere classified |  |
| Outpatient ICD-10 Dx | R06 | abnormalities of breathing |  |
| Outpatient ICD-10 Dx | R22 | localized swelling, mass and lump of skin and subcutaneous tissue |  |
| Outpatient ICD-10 Dx | R26 | abnormalities of gait and mobility |  |
| Outpatient ICD-10 Dx | R53 | malaise and fatigue |  |
| Outpatient ICD-10 Dx | R60 | edema, not elsewhere classified |  |
| Outpatient ICD-10 Dx | R73 | elevated blood glucose level |  |
| Outpatient ICD-10 Dx | R91 | abnormal findings on diagnostic imaging of lung |  |
| Outpatient ICD-10 Dx | Z00 | encounter for general examination without complaint, suspected or reported diagnosis |  |
| Outpatient ICD-10 Dx | Z48 | encounter for other postprocedural aftercare |  |
| Outpatient ICD-10 Dx | Z51 | encounter for other aftercare and medical care |  |
| Outpatient ICD-10 Dx | Z79 | long term (current) drug therapy |  |
| Outpatient ICD-10 Dx | Z86 | personal history of certain other diseases |  |
| Outpatient ICD-10 Dx | Z91 | personal risk factors, not elsewhere classified |  |
| Outpatient ICD-10 Dx | Z95 | presence of cardiac and vascular implants and grafts |  |
| Outpatient ICD-10 Dx | Z99 | dependence on enabling machines and devices, not elsewhere classified |  |
| Outpatient CPT-4 Px | 11721 | debride nail, 6 or more |  |
| Outpatient CPT-4 Px | 17000 | destroy premalg lesion |  |
| Outpatient CPT-4 Px | 20610 | drain/inject, joint/bursa |  |
| Outpatient CPT-4 Px | 36415 | routine venipuncture | Y |
| Outpatient CPT-4 Px | 36416 | capillary blood draw |  |
| Outpatient CPT-4 Px | 70450 | ct head/brain w/o dye |  |
| Outpatient CPT-4 Px | 71010 | chest x-ray |  |
| Outpatient CPT-4 Px | 71020 | chest x-ray |  |
| Outpatient CPT-4 Px | 73030 | x-ray shoulder |  |
| Outpatient CPT-4 Px | 76499 | radiographic procedure |  |
| Outpatient CPT-4 Px | 77052 | comp screen mammogram add-on | Y |
| Outpatient CPT-4 Px | 78452 | myocardial perfusion imaging, tomographic |  |
| Outpatient CPT-4 Px | 78465 | heart image (3d), multiple |  |
| Outpatient CPT-4 Px | 78478 | heart wall motion add-on |  |
| Outpatient CPT-4 Px | 78480 | heart function add-on |  |
| Outpatient CPT-4 Px | 80048 | metabolic panel total ca |  |
| Outpatient CPT-4 Px | 80050 | general health panel |  |
| Outpatient CPT-4 Px | 80053 | comprehen metabolic panel |  |
| Outpatient CPT-4 Px | 80061 | lipid panel |  |
| Outpatient CPT-4 Px | 81001 | urinalysis, auto w/scope | Y |
| Outpatient CPT-4 Px | 81003 | urinalysis, auto, w/o scope |  |
| Outpatient CPT-4 Px | 82043 | microalbumin, quantitative | Y |
| Outpatient CPT-4 Px | 82306 | assay vitamin d | Y |
| Outpatient CPT-4 Px | 82550 | assay ck (cpk) | Y |
| Outpatient CPT-4 Px | 82570 | assay urine creatinine |  |
| Outpatient CPT-4 Px | 82607 | vitamin b-12 |  |
| Outpatient CPT-4 Px | 82728 | assay ferritin |  |
| Outpatient CPT-4 Px | 82746 | blood folic acid serum |  |
| Outpatient CPT-4 Px | 82962 | glucose blood test |  |
| Outpatient CPT-4 Px | 83036 | glycosylated hemoglobin test | Y |
| Outpatient CPT-4 Px | 83540 | assay iron |  |
| Outpatient CPT-4 Px | 83550 | iron binding test |  |
| Outpatient CPT-4 Px | 83735 | assay magnesium |  |
| Outpatient CPT-4 Px | 83880 | natriuretic peptide |  |
| Outpatient CPT-4 Px | 83970 | assay parathormone |  |
| Outpatient CPT-4 Px | 84153 | assay psa, total | Y |
| Outpatient CPT-4 Px | 84439 | assay free thyroxine | Y |
| Outpatient CPT-4 Px | 84443 | assay thyroid stim hormone | Y |
| Outpatient CPT-4 Px | 84484 | assay troponin, quant |  |
| Outpatient CPT-4 Px | 84550 | assay blood/uric acid |  |
| Outpatient CPT-4 Px | 85018 | hemoglobin |  |
| Outpatient CPT-4 Px | 85025 | complete cbc w/auto diff wbc |  |
| Outpatient CPT-4 Px | 85027 | complete cbc, automated |  |
| Outpatient CPT-4 Px | 85610 | prothrombin time |  |
| Outpatient CPT-4 Px | 85730 | thromboplastin time, partial |  |
| Outpatient CPT-4 Px | 88142 | cytopath, c/v, thin layer |  |
| Outpatient CPT-4 Px | 88175 | cytopath c/v auto fluid redo |  |
| Outpatient CPT-4 Px | 88305 | tissue exam by pathologist | Y |
| Outpatient CPT-4 Px | 89240 | pathology lab procedure | Y |
| Outpatient CPT-4 Px | 90658 | flu vaccine, 3 yrs & >, im |  |
| Outpatient CPT-4 Px | 92014 | eye exam & treat | Y |
| Outpatient CPT-4 Px | 92015 | refraction |  |
| Outpatient CPT-4 Px | 93000 | ecg complete | Y |
| Outpatient CPT-4 Px | 93005 | ecg tracing |  |
| Outpatient CPT-4 Px | 93010 | ecg report |  |
| Outpatient CPT-4 Px | 93015 | cardiovascular stress test |  |
| Outpatient CPT-4 Px | 93016 | cardiovascular stress test |  |
| Outpatient CPT-4 Px | 93018 | cardiovascular stress test |  |
| Outpatient CPT-4 Px | 93306 | echocardiography, transthoracic, real-time with image documentation |  |
| Outpatient CPT-4 Px | 93307 | echo exam heart |  |
| Outpatient CPT-4 Px | 93880 | extracranial study |  |
| Outpatient CPT-4 Px | 94060 | evaluate wheezing |  |
| Outpatient CPT-4 Px | 96372 | therapeutic, prophylactic, or diagnostic injection |  |
| Outpatient CPT-4 Px | 97100 | **therapeutic exercises, performed by the patient** |  |
| Outpatient CPT-4 Px | 98941 | chiropractic manipulation |  |
| Outpatient CPT-4 Px | 99204 | office/outpatient visit, new | Y |
| Outpatient CPT-4 Px | 99212 | office/outpatient visit, est |  |
| Outpatient CPT-4 Px | 99213 | office/outpatient visit, est | Y |
| Outpatient CPT-4 Px | 99214 | office/outpatient visit, est |  |
| Outpatient CPT-4 Px | 99215 | office/outpatient visit, est |  |
| Outpatient CPT-4 Px | 99244 | office consultation | Y |
| Outpatient CPT-4 Px | 99245 | office consultation |  |
| Outpatient CPT-4 Px | 99283 | emergency dept visit |  |
| Outpatient CPT-4 Px | 99284 | emergency dept visit |  |
| Outpatient CPT-4 Px | 99285 | emergency dept visit |  |
| Outpatient CPT-4 Px | 99291 | critical care, first hour |  |
| Outpatient CPT-4 Px | 99396 | prev visit, est, age 40-64 |  |
| Outpatient CPT-4 Px | A0425 | ground mileage, per statute mile |  |
| Outpatient CPT-4 Px | A0427 | ambulance service, advanced life support, emergency transport |  |
| Outpatient CPT-4 Px | A4253 | blood glucose test or reagent strips for home blood glucose monitor |  |
| Outpatient CPT-4 Px | A4256 | normal, low and high calibrator solution / chips |  |
| Outpatient CPT-4 Px | A4259 | lancets |  |
| Outpatient CPT-4 Px | A9500 | technetium tc-99m sestamibi, diagnostic, per study dose |  |
| Outpatient CPT-4 Px | E1390 | oxygen concentrator, single delivery port, capable of delivering 85 percent or greater oxygen concentration at the prescribed flow rate |  |
| Outpatient CPT-4 Px | G0001 | routine venipuncture for collection of specimen |  |
| Outpatient CPT-4 Px | G0008 | admin influenza virus vac |  |
| Outpatient CPT-4 Px | G0009 | admin pneumococcal vac |  |
| Outpatient CPT-4 Px | G0202 | Screening mammography, bilateral |  |
| Outpatient CPT-4 Px | J1030 | injection, methylprednisolone acetate, 40 mg |  |
| Outpatient CPT-4 Px | J2250 | injection, midazolam hydrochloride, per 1 mg |  |
| Outpatient CPT-4 Px | J3010 | injection, fentanyl citrate, 0.1 mg |  |
| Outpatient CPT-4 Px | J3301 | injection, triamcinolone acetonide, not otherwise specified, 10 mg |  |
| Outpatient CPT-4 Px | Q9967 | low osmolar contrast material, 300-399 mg/ml iodine concentration, per ml |  |
| Other Setting ICD-9 Dx | 110 | dermatophytosis |  |
| Other Setting ICD-9 Dx | 250 | diabetes mellitus |  |
| Other Setting ICD-9 Dx | 285 | anemia nec/nos |  |
| Other Setting ICD-9 Dx | 296 | affective psychoses |  |
| Other Setting ICD-9 Dx | 401 | essential hypertension |  |
| Other Setting ICD-9 Dx | 428 | heart failure |  |
| Other Setting ICD-9 Dx | 429 | ill-defined heart dis |  |
| Other Setting ICD-9 Dx | 438 | late eff cerebrovasc dis |  |
| Other Setting ICD-9 Dx | 454 | varicose veins |  |
| Other Setting ICD-9 Dx | 486 | pneumonia |  |
| Other Setting ICD-9 Dx | 496 | chr airway obstruct nec |  |
| Other Setting ICD-9 Dx | 578 | gastrointestinal hemorr |  |
| Other Setting ICD-9 Dx | 682 | other cellulitis/abscess |  |
| Other Setting ICD-9 Dx | 728 | dis of muscle/lig/fascia |  |
| Other Setting ICD-9 Dx | 781 | nerv/musculskel sys symp |  |
| Other Setting ICD-9 Dx | 782 | skin/oth integument symp |  |
| Other Setting ICD-9 Dx | 788 | urinary system symptoms |  |
| Other Setting ICD-9 Dx | V55 | atten to artificial open |  |
| Other Setting ICD-9 Dx | V58 | encountr proc/aftrcr nec |  |
| Other Setting ICD-10 Dx | I10 | essential (primary) hypertension |  |
| Other Setting CPT-4 Px | 11721 | debride nail, 6 or more |  |
| Other Setting CPT-4 Px | 36415 | routine venipuncture |  |
| Other Setting CPT-4 Px | 74000 | x-ray abdomen |  |
| Other Setting CPT-4 Px | 99305 | nursing facility care, init |  |
| Other Setting CPT-4 Px | 99306 | nursing facility care, init |  |
| Other Setting CPT-4 Px | 99307 | nursing fac care, subseq |  |
| Other Setting CPT-4 Px | 99308 | nursing fac care, subseq |  |
| Other Setting CPT-4 Px | 99309 | nursing fac care, subseq |  |
| Other Setting CPT-4 Px | Q0092 | set-up portable x-ray equipment |  |
| Other Setting CPT-4 Px | R0070 | transport portable x-ray |  |
| Other Setting CPT-4 Px | R0075 | transport port x-ray multiple |  |
| Laboratory Tests | 10834-0 | globulin [mass/volume] in serum by calculation | Y |
| Laboratory Tests | 11054-4 | cholesterol in ldl/cholesterol in hdl [mass ratio] in serum or plasma |  |
| Laboratory Tests | 11579-0 | thyrotropin [units/volume] in serum or plasma by detection limit <= 0.05 miu/l | Y |
| Laboratory Tests | 12235-8 | microscopic observation [identifier] in urine sediment by light microscopy |  |
| Laboratory Tests | 13945-1 | erythrocytes [#/area] in urine sediment by microscopy high power field |  |
| Laboratory Tests | 14957-5 | microalbumin [mass/volume] in urine | Y |
| Laboratory Tests | 1742-6 | alanine aminotransferase [enzymatic activity/volume] in serum or plasma | Y |
| Laboratory Tests | 1751-7 | albumin [mass/volume] in serum or plasma | Y |
| Laboratory Tests | 1754-1 | albumin [mass/volume] in urine | Y |
| Laboratory Tests | 1759-0 | albumin/globulin [mass ratio] in serum or plasma | Y |
| Laboratory Tests | 17861-6 | calcium [mass/volume] in serum or plasma | Y |
| Laboratory Tests | 1920-8 | aspartate aminotransferase [enzymatic activity/volume] in serum or plasma | Y |
| Laboratory Tests | 1975-2 | bilirubin.total [mass/volume] in serum or plasma | Y |
| Laboratory Tests | 19763-2 | specimen source [identifier] in cervical or vaginal smear or scraping by cyto stain |  |
| Laboratory Tests | 19764-0 | statement of adequacy [interpretation] of cervical or vaginal smear or scraping by cyto stain |  |
| Laboratory Tests | 19767-3 | cytologist who read cyto stain of cervical or vaginal smear or scraping |  |
| Laboratory Tests | 19769-9 | pathologist who read cyto stain of cervical or vaginal smear or scraping |  |
| Laboratory Tests | 19773-1 | recommended follow-up [identifier] in cervical or vaginal smear or scraping by cyto stain |  |
| Laboratory Tests | 1986-9 | c peptide [mass/volume] in serum or plasma |  |
| Laboratory Tests | 2028-9 | carbon dioxide, total [moles/volume] in serum or plasma | Y |
| Laboratory Tests | 2075-0 | chloride [moles/volume] in serum or plasma | Y |
| Laboratory Tests | 2086-7 | deprecated cholesterol.in hdl [mass/volume] in serum or plasma |  |
| Laboratory Tests | 2132-9 | cobalamin (vitamin b12) [mass/volume] in serum or plasma |  |
| Laboratory Tests | 2160-0 | creatinine [mass/volume] in serum or plasma | Y |
| Laboratory Tests | 22636-5 | pathology report relevant history narrative |  |
| Laboratory Tests | 22637-3 | pathology report final diagnosis narrative |  |
| Laboratory Tests | 22638-1 | pathology report comments [interpretation] narrative |  |
| Laboratory Tests | 22639-9 | pathology report supplemental reports narrative |  |
| Laboratory Tests | 2284-8 | folate [mass/volume] in serum or plasma |  |
| Laboratory Tests | 2823-3 | potassium [moles/volume] in serum or plasma | Y |
| Laboratory Tests | 2885-2 | protein [mass/volume] in serum or plasma | Y |
| Laboratory Tests | 2951-2 | sodium [moles/volume] in serum or plasma | Y |
| Laboratory Tests | 3094-0 | urea nitrogen [mass/volume] in serum or plasma | Y |
| Laboratory Tests | 3097-3 | urea nitrogen/creatinine [mass ratio] in serum or plasma | Y |
| Laboratory Tests | 47527-7 | cytology report of cervical or vaginal smear or scraping cyto stain.thin prep |  |
| Laboratory Tests | 48642-3 | glomerular filtration rate/1.73 sq m predicted among non-blacks [volume rate/area] in serum or plasm |  |
| Laboratory Tests | 48643-1 | glomerular filtration rate/1.73 sq m predicted among blacks [volume rate/area] in serum or plasma by | Y |
| Laboratory Tests | 5902-2 | prothrombin time (pt) |  |
| Laboratory Tests | 6301-6 | inr in platelet poor plasma by coagulation assay |  |
| Laboratory Tests | 6463-4 | bacteria identified in unspecified specimen by culture |  |
| Laboratory Tests | 6777-7 | deprecated glucose [mass/volume] in serum or plasma |  |
| Laboratory Tests | 732-8 | lymphocytes [#/volume] in blood by manual count |  |
| Laboratory Tests | 9318-7 | albumin/creatinine [mass ratio] in urine | Y |
| Laboratory Tests | UNLOINC | UNLOINC |  |
| CPT-4 = Current Procedural Terminology; Dx = diagnosis; ICD-9 = International Classification of Diseases, 9th Revision; NDC = National Drug Code; Px = procedure  * In a sensitivity analysis, covariates with z-bias=5 were excluded from the propensity score as they were strongly related to exposure, but not outcome | | | |

**Supplementary Table 6. Results from secondary analyses**

| **Sensitivity Analyses (Pre-specified)** | **Database** | **N** | **aHR**^†^ **for SCA/VA** | | **aHR**^†^ **for SCD/Fatal VA** | | |
| --- | --- | --- | --- | --- | --- | --- | --- |
|  |  |  | **Glimepiride** | **Glyburide** | **Glimepiride** | | **Glyburide** |
| **Re-ran multinomial propensity score model excluding covariates with z-score=5** | **Medicaid** | 624,406 | 1.15 (0.94-1.40) | 0.87 (0.73-1.03) | 1.30 (0.99-1.70) | | 0.90 (0.71-1.14) |
|  | **Optum** | 491,940 | 0.84 (0.65-1.08) | 1.10 (0.86-1.41) | - | | - |
| **Excluded persons with baseline enrollment in Medicaid managed care** | **Medicaid** | 269,109 | 1.18 (0.92-1.51) | 0.90 (0.73-1.12) | 1.52 (1.09-2.13) | | 0.91 (0.67-1.23) |
|  | **Optum** | - | - | - | - | | - |
| **Decreased permissible grace period between contiguous sulfonylurea dispensing from 15 days to 7 days** | **Medicaid** | 624,406 | 1.15 (0.93-1.43) | 0.88 (0.73-1.06) | 1.36 (1.01-1.82) | | 0.91 (0.70-1.18) |
|  | **Optum** | 491,940 | 0.89 (0.67-1.18) | 1.03 (0.77-1.38) | - | | - |
| **Increased permissible grace period between contiguous sulfonylurea dispensing from 15 days to 30 days** | **Medicaid** | 624,406 | 1.09 (0.90-1.31) | 0.89 (0.77-1.04) | 1.33 (1.02-1.75) | | 0.90 (0.71-1.14) |
|  | **Optum** | 491,940 | 0.85 (0.67-1.06) | 1.12 (0.90-1.40) | - | | - |
| **Excluded persons with any-claim type, any position diagnosis of SCA or VA ever prior to cohort entry** | **Medicaid** | 613,858 | 1.18 (0.96-1.45) | 0.88 (0.73-1.04) | 1.38 (1.05-1.83) | | 0.93 (0.73-1.18) |
|  | **Optum** | 488,489 | 0.81 (0.62-1.04) | 1.09 (0.85-1.40) | - | | - |
| **Sensitivity Analyses (Ad Hoc)** | **Database** | **N** | **aHR**^†^ **for SCA/VA** | | **aHR**^†^ **for SCD/Fatal VA** | | |
|  |  |  | **Glimepiride** | **Glyburide** | **Glimepiride** | | **Glyburide** |
| **Re-ran multinomial propensity score model excluding laboratory result covariates** | **Optum** | 491,940 | 0.84 (0.65-1.08) | 1.10 (0.86-1.41) | **-** | | **-** |
| **Re-ran multinomial propensity score model excluding laboratory result and socioeconomic covariates** | **Optum** | 491,940 | 0.83 (0.65-1.07) | 1.11 (0.86-1.42) | **-** | | **-** |
| **Examining Effect Modification by:** | **Population** | **aHR**^†^ **for SCA/VA** | | | **aHR**^†^ **for SCD/Fatal VA** | | |
|  |  | **P-Value for Interaction Term** | **Glimepiride** | **Glyburide** | **P-Value for Interaction Term** | **Glimepiride** | **Glyburide** |
| **CYP2C9 inhibitors** | **Medicaid** | 0.274 | Since the interaction term p-values did not meet the pre-specified threshold for statistical significance, stratified results are not presented | | 0.379 | Since the interaction term p-values did not meet the pre-specified threshold for statistical significance, stratified results are not presented | |
|  | **Optum** | 0.616 |  |  | - | - | - |
| **CYP3A4 inhibitors** | **Medicaid** | 0.215 |  |  | 0.028 | Use of CYP3A4: 4.14 (1.60-10.71)  No use of CYP3A4: 1.20 (0.91-1.60) | Use of CYP3A4: 1.10 (0.37-3.27)  No use of CYP4A4: 0.90 (0.71-1.15) |
|  | **Optum** | 0.427 |  |  | - | - | - |
| **Drugs with “known risk” of TdP** | **Medicaid** | 0.342 |  |  | 0.663 | Since the interaction term p-values did not meet the pre-specified threshold for statistical significance, stratified results are not presented | |
|  | **Optum** | 0.383 | Since the interaction term p-values did not meet the pre-specified threshold for statistical significance, stratified results are not presented | | - | - | - |
| **Drugs with any risk of TdP** | **Medicaid** | 0.472 |  |  | 0.388 | Since the interaction term p-values did not meet the pre-specified threshold for statistical significance, stratified results are not presented | |
|  | **Optum** | 0.198 |  |  | - | - | - |
| **Age Group** | **Medicaid** | 0.087 |  |  | 0.028 | 30-41 years: 1.01 (0.20-4.98)  42-51 years: 3.53 (1.74-7.17)  52-61 years: 0.83 (0.47-1.46)  62-75 years: 1.32 (0.92-1.88) | 30-41 years: 1.59 (0.55-4.57)  42-51 years: 0.87 (0.39-1.97)  52-61 years: 0.84 (0.55-1.30)  62-75 years: 0.91 (0.67-1.24) |
|  | **Optum** | 0.665 |  |  | - | - | - |
| **Sex** | **Medicaid** | 0.977 |  |  | 0.325 | Since the interaction term p-values did not meet the pre-specified threshold for statistical significance, stratified results are not presented | |
|  | **Optum** | 0.999 | Since the interaction term p-values did not meet the pre-specified threshold for statistical significance, stratified results are not presented | | - | - | - |
| **Race** | **Medicaid** | 0.566 |  |  | 0.378 | Since the interaction term p-values did not meet the pre-specified threshold for statistical significance, stratified results are not presented | |
|  | **Optum** | 0.218 |  |  | - | - | - |
| **Nursing Home Residence** | **Medicaid** | 0.187 |  |  | 0.317 | Since the interaction term p-values did not meet the pre-specified threshold for statistical significance, stratified results are not presented | |
|  | **Optum** | 0.234 |  |  | - | - | - |
| **Ischemic Heart Disease** | **Medicaid** | 0.520 |  |  | 0.350 | Since the interaction term p-values did not meet the pre-specified threshold for statistical significance, stratified results are not presented | |
|  | **Optum** | 0.030 | Ischemic Heart Disease: 0.62 (0.42-0.94)  No Ischemic Heart Disease: 1.02 (0.74-1.40) | Ischemic Heart Disease: 1.31 (0.91-1.89)  No Ischemic Heart Disease: 1.00 (0.72-1.39) | - | - | - |
| **Conduction Disorders** | **Medicaid** | 0.516 | Since the interaction term p-values did not meet the pre-specified threshold for statistical significance, stratified results are not presented | | 0.960 | Since the interaction term p-values did not meet the pre-specified threshold for statistical significance, stratified results are not presented | |
|  | **Optum** | 0.389 |  |  | - | - | - |
| **HF/Cardiomyopathy** | **Medicaid** | 0.466 |  |  | 0.925 | Since the interaction term p-values did not meet the pre-specified threshold for statistical significance, stratified results are not presented | |
|  | **Optum** | 0.801 |  |  | - | - | - |
| **Kidney Disease** | **Medicaid** | 0.928 |  |  | 0.034 | Kidney Disease: 1.97 (1.31-2.97)  No Kidney Disease: 0.99 (0.69-1.43) | Kidney Disease: 1.21 (0.81-1.81)  No Kidney Disease: 0.80 (0.60-1.06) |
|  | **Optum** | 0.052 |  |  | - | - | - |
| aHR = adjusted hazard ratio; CYP = cytochrome P450; HF = heart failure; N = number of second-generation sulfonylurea users under study; SCA = sudden cardiac arrest; SCD = sudden cardiac death; TdP = torsade de pointes; VA = ventricular arrhythmia  † Vs. glipizide as pre-specified referent | | | | | | | |

**Supplementary Table 7. Results from dose-response analyses**

| **Second-Generation Sulfonylurea** | **Average Daily Dose (mg)** | **aHR for SCA/VA** | |
| --- | --- | --- | --- |
|  |  | **Medicaid** | **Optum** |
| **Glipizide, immediate release** | **0-2.5** | 0.94 (0.55-1.62) | 1.23 (0.42-3.59) |
|  | **> 2.5-5** | 1.00 (referent) | 1.00 (referent) |
|  | **> 5-10** | 1.06 (0.74-1.51) | 0.91 (0.47-1.74) |
|  | **> 10** | 0.65 (0.40-1.06) | 1.71 (0.88-3.35) |
| **Glipizide, extended release** | **0-2.5** | 0.78 (0.42-1.44) | 1.43 (0.66-3.10) |
|  | **> 2.5-5** | 1.00 (referent) | 1.00 (referent) |
|  | **> 5-10** | 1.06 (0.59-1.90) | 1.19 (0.53-2.67) |
|  | **> 10** | 0.55 (0.19-1.60) | 0.67 (0.15-3.04) |
| **Glyburide, non-micronized** | **0-1.5** | 0.61 (0.26-1.39) | 1.33 (0.39-4.53) |
|  | **> 1.5-5** | 1.00 (referent) | 1.00 (referent) |
|  | **> 5-10** | 0.98 (0.67-1.43) | 1.53 (0.83-2.86) |
|  | **> 10** | 1.09 (0.67-1.78) | 1.09 (0.46-2.56) |
| **Glimepiride** | **< 1** | 1.88 (0.66-5.37) | **-** |
|  | **1-2** | 1.00 (referent) | **-** |
|  | **≤ 2** | - | 1.00 (referent) |
|  | **> 2-4** | 0.71 (0.43-1.17) | 0.98 (0.54-1.77) |
|  | **> 4** | 0.92 (0.48-1.76) | 0.89 (0.37-2.13) |
| aHR = adjusted hazard ratio; mg = milligrams; SCA = sudden cardiac arrest; VA = ventricular arrhythmia | | | |

**Supplementary Table 8. Operational definition for composite outcome of interest**

| **Outcome component** | **Diagnosis** | **ICD-9-CM discharge diagnosis code*** | **ICD-10-CM discharge diagnosis code**** | **Discharge diagnosis position and claim type** |
| --- | --- | --- | --- | --- |
| sudden cardiac arrest | cardiac arrest | 427.5 | I46.2  I46.8  I46.9 | First-listed discharge diagnosis on an emergency department claim or a principal discharge diagnosis on an inpatient hospitalization claim |
|  | sudden death, cause unknown | 798 | - |  |
|  | instantaneous death | 798.1 | - |  |
|  | death occurring in less than 24 hours from onset of symptoms, not otherwise explained | 798.2 | - |  |
| ventricular arrhythmia | (paroxysmal) ventricular tachycardia | 427.1 | I47.2 |  |
|  | ventricular fibrillation and flutter | 427.4 | I49.0 |  |
|  | ventricular fibrillation | 427.41 | I49.01 |  |
|  | ventricular flutter | 427.42 | I49.02 |  |
| CM = Clinical Modification; ICD = International Classification of Diseases  *ICD-9-CM codes used for January 1, 1999 through December 31, 2012 in Medicaid data and January 1, 2000 through September 30, 2015 in Optum data  **ICD-10-CM codes used for October 1, 2015 through December 31, 2016 in Optum data; the conversion to ICD-10-CM from ICD-9-CM was achieved via forward-backward general equivalence mapping^1^ and subsequently vetted by a Penn Medicine cardiac electrophysiologist  1. Fung et al. Preparing for the ICD-10-CM transition: Automated methods for translating ICD codes in clinical phenotype definitions. *EGEMS (Wash DC)* 2016;4(1):1211. | | | | |
